# Lipoprotein *N*-terminal modification in *Bacillus*: a new paradigm for extracellular acetylation and species-dependent Toll-like receptor 2 immunomodulation

**DOI:** 10.1128/mbio.00996-25

**Published:** 2025-07-08

**Authors:** Gloria Komazin, Rachel M. Wigmore, Aditi M. Ranade, Amena A. Rizk, John H. Gardiner, Timothy C. Meredith

**Affiliations:** 1 Department of Biochemistry and Molecular Biology, The Pennsylvania State University 189499, University Park, Pennsylvania, USA; 2 The Huck Institutes of the Life Sciences, The Pennsylvania State University 124474, University Park, Pennsylvania, USA; University of Georgia, Atlanta, Georgia, USA; Emory University School of Medicine, Atlanta, Georgia, USA

**Keywords:** bacterial lipoproteins, acetyltransferase, Toll-like receptor 2, acylation, extracellular acetylation

## Abstract

Lipoproteins are a defining feature of extracellular bacterial membrane surfaces. Acylation of an invariant *N*-terminal cysteine residue tethers proteins to the cell surface, where they participate in many cellular processes occurring at the membrane-environment interface. Lipoproteins also double as key ligands for bacterial detection by the mammalian innate immune system, which when bound by Toll-like receptor 2 (TLR2) complexes orchestrate a pro-inflammatory response to clear infections. While nearly all bacteria synthesize lipoproteins, variable acylation states can attenuate TLR2 signaling. In *Bacillus subtilis*, lipoproteins are *N*-acetylated to form acetylated lipoproteins (Ac-LP) after being transported across the membrane. How high-energy acetyl donors are shuttled to the cell surface for trasnfer is unknown. Using a transposon-based genetic screen to identify mutants with altered TLR2 activation and *in vitro* reconstitution of the enzyme machinery, we now describe an acetylated heptaprenylglyceryl (Ac-HepG) carrier synthesized by PcrB/YvoF in the cytosol that is required for lipoprotein acetylation. We propose Ac-HepG is shuttled to the outer membrane leaflet for use by the previously uncharacterized integral membrane protein, lipoprotein heptaprenylglyceryl *N*-acetyl transferase (LhaT) (formerly YpjA), for *N*-acetylation of the α-amino termini of lipoprotein substrates. We provide evidence that LhaT, which in most *Bacillus* spp. makes the high-affinity Ac-LP TLR2 ligand, has become pseudogenized in the *Bacillus cereus* group subclade that harbors opportunistic pathogens. A naturally occurring poly-tyrosine insertion within LhaT from *Bacillus anthracis* isolates prevents acetyl transfer, allowing the flanking lipoprotein remodeling gene *lit* to quantitatively convert the population to the TLR2-silent lyso-lipoprotein (lyso-LP) chemotype.

**IMPORTANCE:**

Protein acetylation is an important and widespread post-translational modification. Annotation of LhaT and the lipoprotein *N*-acetylation pathway provides a paradigm for how cells can source high-energy extracellular acetyl donors, with the enigmatic lipid Ac-HepG now joining the cytosolic acetyl-coenzyme A and acetyl-phosphate as acetyl group donors. While Ac-LP biosynthetic pathway genes appear to be universally conserved in all *Bacillus* spp., those associated with pathogenic lineages have often lost function. In *B. anthracis* strains, LhaT has been inactivated by insertion of poly-tyrosine runs of variable length that favors chemotype conversion to lyso-LP and evasion of TLR2 detection. Lipoprotein chemotypes are thus critical determinants governing environmental and pathogen differentiation among *Bacillus* spp.

## INTRODUCTION

Lipoproteins are ubiquitous cell envelope proteins in both gram-positive and gram-negative bacteria, encoded by 2%–5% of all genes in a typical genome (1–4). Serving key functions in nearly every aspect of bacterial cell envelope physiology, lipoproteins bind extracellular carbohydrates/ions/amino acid nutrients, help fold and chaperone secreted proteins, direct adhesion to cells and surfaces, act as structural components, and tether virulence factors. Post-translational acylation of an invariant *N*-terminal cysteine residue is required for membrane surface retention. Lipoproteins are first translated in the cytosol as pre-prolipoproteins with an *N*-terminal export signal peptide preceding a “lipobox” consensus sequence. Once on the extracytoplasmic surface of the membrane, the initial lipoprotein pathway enzyme lipoprotein diacylglyceryl transferase (Lgt) forms a thioether through nucleophilic displacement of the headgroup of a diacylglyceryl phospholipid donor (5). The signal peptide is then removed by a signal peptide II-specific protease (Lsp) to liberate the α-amino group of cysteine and form diacylglyceryl lipoprotein (DA-LP) (6, 7). While the early steps in lipoprotein biosynthesis are well conserved in both diderm gram-negative and monoderm gram-positive bacteria, the majority of gram-negative organisms go on to make triacylated lipoprotein (TA-LP) using genera-specific lipoprotein *N*-acyl transferases (Lnt or Lnb) (8–10), an important step for efficient lipoprotein trafficking to the outer membrane (11, 12).

Despite the notable absence of *N*-acyl transferase gram-negative orthologs in monoderm Firmicutes genomes, many do further tailor the α-amino DA-LP terminus using specific enzymes and regulatory controls that can vary at both the genera and species levels (13). In *Enterococcus faecalis*, the lipoprotein intramolecular transferase (Lit) shifts an *O*-linked acyl chain ester from *SN*_2_ of the glyceryl unit to the α-amino DA-LP terminus to form the lyso-lipoprotein (lyso-LP) chemotype (14), while a two-enzyme acylation system (LnsAB) forms TA-LP in *Staphylococcus aureus* (15). Structural divergence and selection for a particular lipoprotein chemotype appear to at least in part be driven by mammalian innate immunity, which uses the unique *N*-terminal acylation pattern as a focal point for bacterial detection through Toll-like receptor 2 (TLR2) family signaling (16, 17). Binding of lipoproteins to TLR2 complexes expressed on the surface of macrophages and other immunosurveillance cell types triggers pro-inflammatory cytokine/chemokine secretion to help clear invading bacteria, as well as to orchestrate humoral immunity (18). To accommodate *N*-terminal structural variation among bacterial lipoproteins, TLR2 family receptors form chemotype-specific heterodimeric complexes. The TLR2/1 complex binds *N*-acylated variants, while TLR2/6 recognizes free α-amino and short chain variants such as *N*-acetylated lipoprotein (Ac-LP) (13). Lyso-LP is an extremely weak ligand for either of the canonical TLR2 hetero complexes, with TLR2 detection being 100- to 1,000-fold less sensitive in comparison to isogenic Δ*lit* negative strains that produce DA-LP (19).

Herein we report the identification and reconstitution of the lipoprotein *N*-acetylation pathway in the model Firmicute *Bacillus subtilis*. The multistep pathway requires at minimum three distinct enzymes located at three separate genetic loci to convert the α-amino terminus of DA-LP to Ac-LP. To source the extracellular high-energy acetyl-donor for DA-LP substrate, a heptaprenylglyceryl carrier lipid pre-loaded with acetyl groups from cytosolic acetyl-CoA is proposed to shuttle acetyl units to the previously unknown integral membrane protein, lipoprotein heptaprenylglyceryl *N*-acetyl transferase (LhaT) (formerly YpjA). We provide evidence that the gene encoding LhaT, although seemingly retained in all *Bacillus* spp., is non-functional in *Bacillus anthracis* and other closely related lineages with pathogenic potential due to lyso-LP forming *lit* gene integration and pseudogenization of *lhaT*’.

## RESULTS

### Co-expression of lyso- and Ac-LP suppresses TLR2 signaling

**Fig 1:**
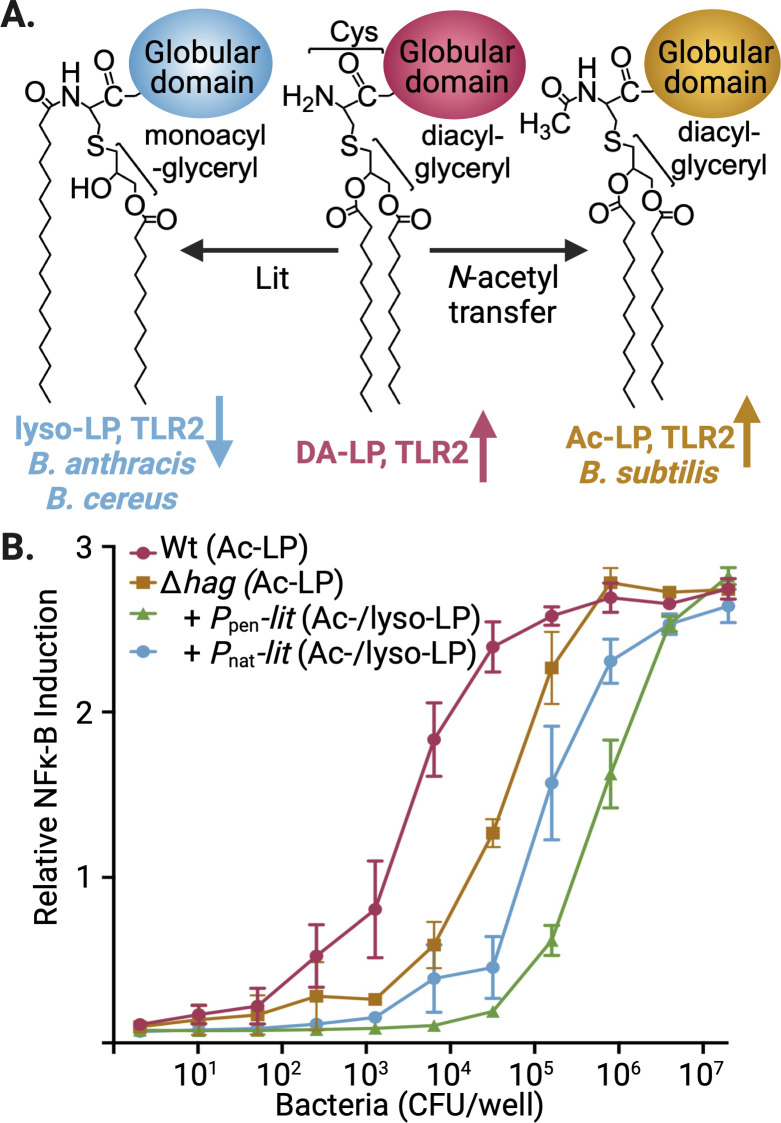
Biosynthesis and TLR2 activation by Ac-LP and lyso-LP chemotypes in *Bacillus* sp. (A) *N*-terminal lipoprotein tailoring pathways for lyso-LP (*B. anthracis* and *B. cereus*) and Ac-LP (*B. subtilis*) chemotypes modify a common free α-amino containing DA-LP substrate. Lyso-LP formed by Lit through intramolecular migration of the *SN*_2_ acyl chain produces a weak TLR2 ligand (↓), while *N-*acetylation to form Ac-LP by an unknown process does not significantly change the high intrinsic TLR2 (↑) activity of DA-LP. (B) Heat-killed aliquots of *B. subtilis* cultures were serially diluted and applied to HEK-Blue hTLR2 reporter cells encoding NF-κB-inducible secreted alkaline phosphatase. Data represent the average of three biological replicates for *B. subtilis* wild type (Wt, Ac-LP), with the flagella deleted (GKM1755 Δ*hag*, Ac-LP) and with heterologous expression of *lit* from *B. cereus* using either the native (TXM1762, Δ*hag P*_nat_-*lit*, Ac-LP/lyso-LP mixed chemotype population) or a strong (GKM1757, Δ*hag P*_pen_-*lit*, Ac-LP/lyso-LP mixed chemotype population) promoter.

To identify genes required for the *N*-acetylation of lipoproteins in *B. subtilis*, we first tested whether introducing the lipoprotein intramolecular acyl transferase *lit* gene from a *Bacillus cereus* isolate would suppress TLR2 signaling (Fig. 1A). Lit remodels lipoproteins by transferring the *SN*_2_ acyl chain of DA-LP to the α-amino terminus, making lyso-LP (4, 14, 20). Unlike DA-LP and Ac-LP, which are inherently strong TLR2/6 agonists, lyso-LP is a very weak TLR2/6 ligand with negligible TLR2/1 affinity (19). We thus hypothesized that co-expression of genes encoding the lyso- and Ac-LP forming pathways in *B. subtilis* would decrease TLR2 signaling. This partial decrease would become complete, however, upon transposon (Tn)-mediated disruption of genes from the Ac-LP pathway since the competition for the DA-LP substrate would be relieved and shift the entire lipoprotein population to the TLR2 silent lyso-LP ligand. Total suppression of TLR2 signaling would thereby offer a convenient assay handle for phenotypic screening using HEK cells transfected with TLR2 and an inducible secreted alkaline phosphatase (SEAP) reporter gene under the control of the TLR2-responsive NF-κB transcription factor. To minimize interfering cross signals from TLR5-mediated detection of flagella subunits which also activate NF-κB (21), the *hag* gene (H antigen) was first deleted in *B. subtilis* before introducing *lit* under the control of either its own native promoter in *B. cereus* (*P*_nat_) or a constitutive strong promoter (*P*_pen_). TLR2 signaling was robustly suppressed with *P*_pen_-driven expression of *lit* but only 5- to 10-fold with *P*_nat_-*lit*, suggesting that a strong TLR2 activating Ac-LP population remained (Fig. 1B). Lipoproteins were extracted and separated by SDS-PAGE, with the structure of more abundant lipoproteins determined by matrix-assisted laser desorption/ionization time-of-flight mass spectrometry (MALDI-TOF MS). Detailed analysis of a representative lipoprotein (desferrioxamine/ferrichrome-binding transporter lipoprotein FrxB) confirmed a mixed chemotype population in the *P*_nat_-*lit* construct (Fig. S1), with a substantial Ac-LP pool subject to shifting to lyso-LP upon disruption of the Ac-LP pathway.

### Deletion of PcrB, YvoF, or YpjA prevents lipoprotein *N*-acetylation

**Fig 2:**
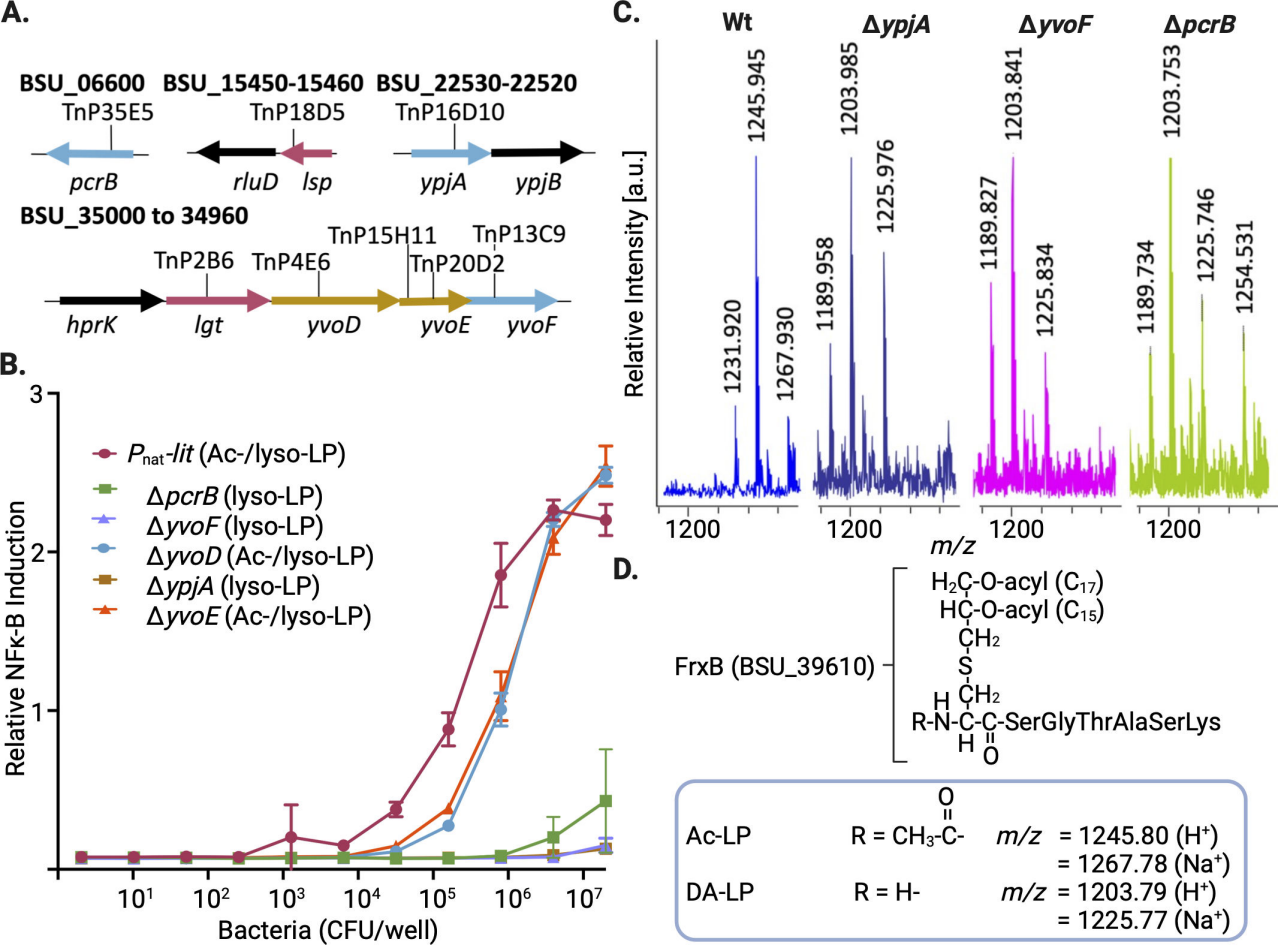
Identification of genes required for Ac-LP formation in *B. subtilis*. (A) A transposon library built in strain TXM1762 (Δ*hag P*_nat_-*lit*) and expressing a mixed Ac-LP/lyso-LP population was screened for loss of TLR2-stimulating activity. Eight mutants with unique Tn insertion sites that mapped to four genetic loci had decreased TLR2 activity. The TLR2 activity could be restored by removing the *P*_nat_-*lit* cassette in all but the *lgt::Tn* mutant background (see Fig. S3). (B) The response of HEK-Blue hTLR2 reporter cells to *B. subtilis* heat-killed extracts was measured in non-polar targeted gene::FRT deletion backgrounds expressing the *P*_nat_-*lit* cassette (TXM1762) for each of the LP *N*-acetylation candidates (TXM1915 [Δ*pcrB*], TXM1916 [Δ*yvoE*)] TXM1917 [Δ*yvoF*], TXM1918 [Δ*yvoD*], and TXM1939 [Δ*ypjA*]) identified in panel A. Data represent the average of three biological replicates. (C) MALDI-TOF MS spectra for targeted gene::FRT deletions in wild-type *B. subtilis* (TXM1938 [Δ*ypjA*], GKM1834 [Δ*yvoF*], and GKM1835 [Δ*pcrB*]) were obtained for *N*-terminal tryptic lipopeptides of the desferrioxamine/ferrichrome-binding transporter lipoprotein FrxB. Masses consistent with conversion to DA-LP (decrease in *m*/*z* by 42 Da) were observed for Δ*pcrB*, Δ*ypjA*, and Δ*yvoF*, while Ac-LP remained the dominant chemotype in spectra for Δ*yvoD* and Δ*yvoE* (see Fig. S4B). Back complementation of *pcrB*/*ypjA*/*yvoF* restored Ac-LP synthesis in all cases (see Fig. S4A). (D) The structure and calculated mass peak assignments for the Ac-LP and DA-LP forms of the *N*-terminus of the FrxB lipopeptide are shown for both the protonated and sodiated adducts. Genes harboring Tn insertions in panel A were deemed required for Ac-LP synthesis (blue shading), required for lipoprotein synthesis (red shading), or non-essential/redundant/polar (mustard shading).

A Tn library was built in the *B. subtilis* Δ*hag P*_nat_-*lit* strain TXM1762 using the C9 HMAR mariner transposase (22, 23). Heat-killed bacterial extracts of Tn mutants were screened for diminished TLR2 signaling (Fig. S2), with Tn mutants exhibiting at minimal a 10-fold lower signal than GKM1757 (*lit* expressed using the strong *P*_pen_ promoter, Fig. 1B) selected for follow-up confirmation. Validated Tn insertion sites conferring low TLR2 activity mapped to four distinct genetic loci (Fig. 2A). Disrupting either Lgt or Lsp, two conserved early steps in lipoprotein biosynthesis (1–3), decreased TLR2 signal as would be expected since the entire pathway is blocked. However, Tn insertion in either the monocistronic open reading frame *pcrB*, in *ypjA*, or in the polycistronic *hprK-lgt-yvoD-yvoE-yvoF* gene cluster also reduced TLR2 signal. To differentiate a shift to all lyso-LP from simply a drop in the total lipoprotein levels (as in *lgt* and *lsp* insertion mutants), TLR2 stimulation was remeasured in Tn mutants from which the *lit* cassette had been removed (Fig. S3). The TLR2 activity was restored in all cases except for the *lgt*::Tn insertion, indicating normal levels of lipoprotein TLR2 agonists. Since some of the Tn hits were within polycistronic operons, targeted mutations using FLP recombination to remove antibiotic resistance selection markers and leave a minimal FRT DNA scar sequence (24) were made to minimize polar effects on neighboring genes before retesting competition with the *P*_nat_-*lit* cassette for DA-LP substrate (Fig. 2B). Only *pcrB*, *yvoF*, and *ypjA* fully suppressed TLR2 activity, indicating these genes are strictly required for Ac-LP synthesis. By contrast, *yvoD* and *yvoE* deletions both retained substantial TLR2-stimulating capacity. Since TLR2 activity cannot effectively discriminate between Ac-LP/DA-LP chemotypes, all five Ac-LP biosynthesis pathway gene candidates were transferred to a wild-type *B. subtilis* background, and the structure of the *N*-terminus of FrxB was characterized by matrix-assisted laser desorption/ionization time-of-flight (MALDI-TOF) analysis. Consistent with the DA-LP substrate competition and TLR2 activity assays, tryptic peptide peak masses corresponding to the *N*-termini decreased by an acetyl unit (minus 42 Da) after *pcrB*, *yvoF*, or *ypjA* deletion (Fig. 2C and D). Back complementation restored wild-type lipopeptide masses in all three cases (Fig. S4A). No mass shifts were observed in *yvoD* and *yvoE* deletion mutants, indicating these genes are not absolutely required for Ac-LP biosynthesis in cells (Fig. S4B). The *yvoD* and *yvoE* genes encode a putative membrane protein and a phosphatase, respectively, and are located upstream of the final gene in the operon *yvoF* (Fig. 2A). Since all of these genes share a common promoter positioned in front of *hprK* (25), *yvoD* and *yvoE* Tn insertion hits were likely false hits arising from negative polar effects on *yvoF*. However, a non-essential or redundant role in Ac-LP synthesis cannot be ruled out.

### Reconstitution of the lipoprotein *N*-acetylation pathway from *B. subtilis*

**Fig 3:**
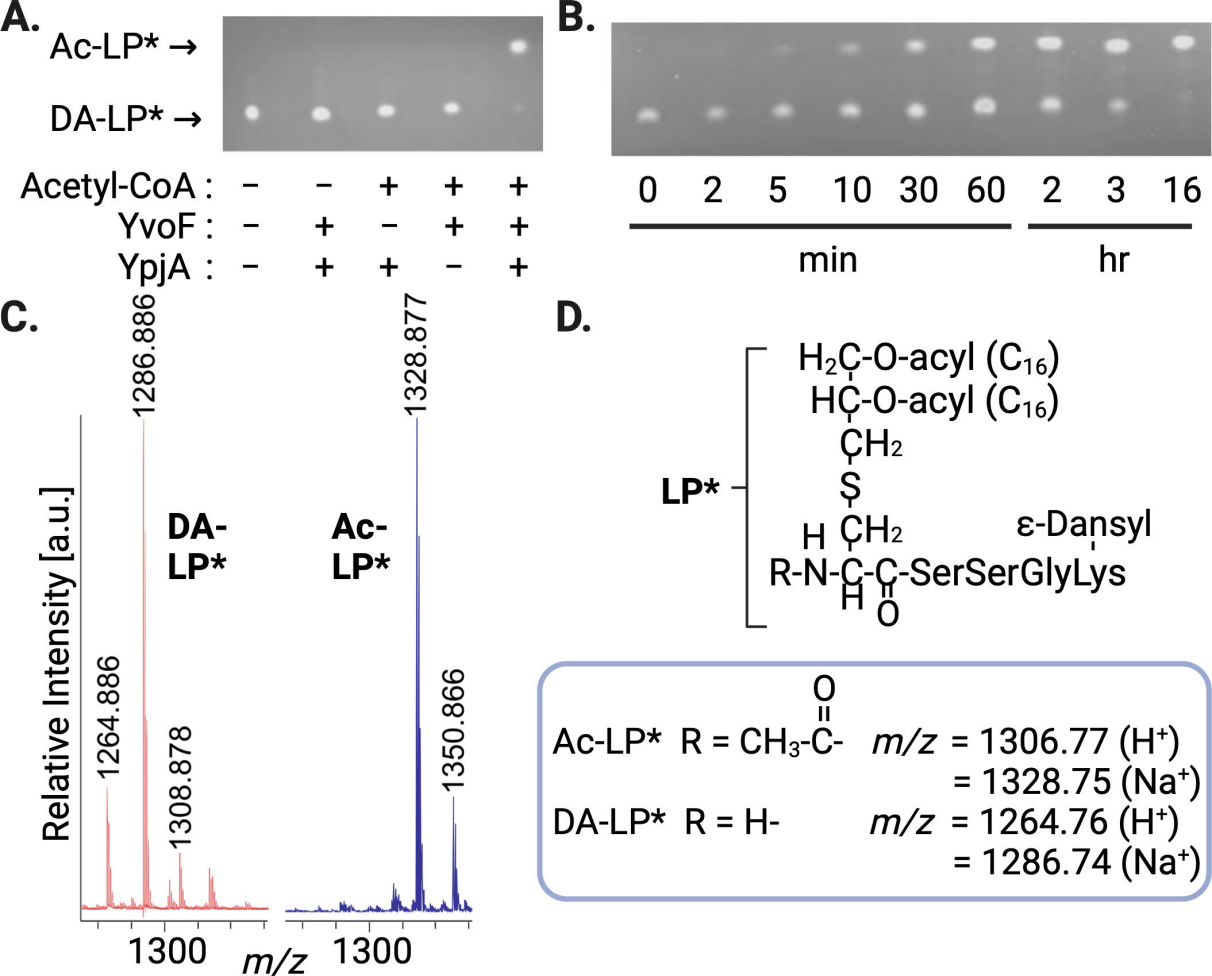
Reconstitution of the lipoprotein *N*-acetylation pathway. (A) Acetylated geranylgeranyl glycerol donor generated *in situ* using geranylgeranyl pyrophosphate, glycerol 1-phosphate, acetyl-CoA, and recombinant PcrB/calf alkaline phosphatase (CIP)/YvoF/YpjA enzymes (see Fig. S5) was mixed with a dansyl-labeled model lipopeptide substrate (*di*-palmitoyl glyceryl-CSSGK, DA-LP*) and incubated for 16 h. Products were separated by TLC and visualized under UV light (365 nm). (B) Reactions were assembled as in panel A and quenched by flash freezing at the indicated time points. (C) Spots corresponding to DA-LP* substrate and Ac-LP* products were scraped from TLC plates and analyzed by MALDI-TOF MS. (D) Calculated masses for both the protonated (H^+^) and sodiated (Na^+^) adducts of DA-LP* and Ac-LP* are shown.

**Fig 4:**
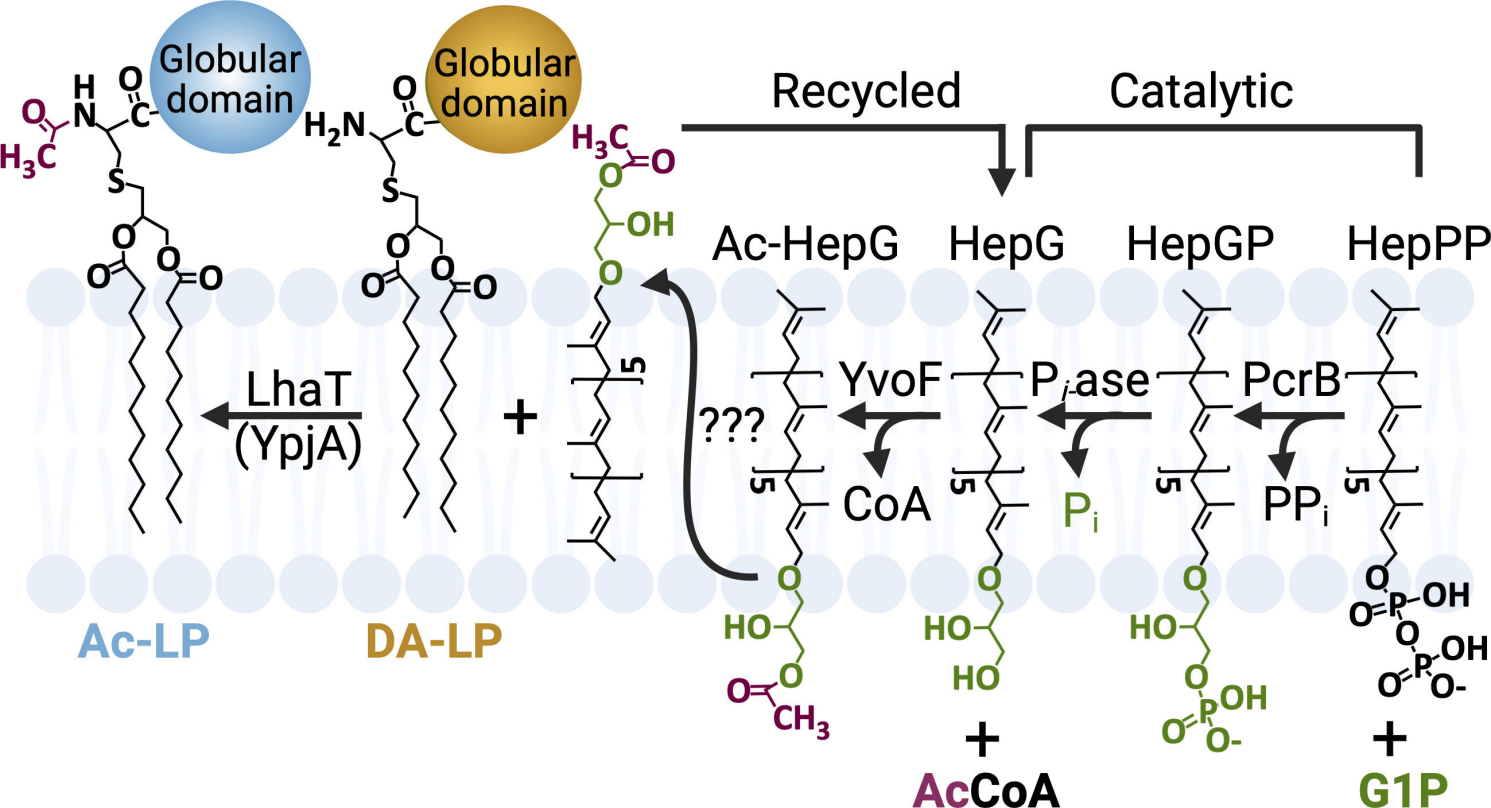
Lipoprotein *N*-acetylation in *B. subtilis* utilizes a heptaprenylglyceryl carrier to shuttle high-energy acetyl donor to the extracellular side of the membrane. Heptaprenyl pyrophosphate (HepPP) and *sn*-glycerol 1-phosphate (G1P) are condensed by PcrB and dephosphorylated by a non-specific phosphatase to make heptaprenyl glycerol (HepG), which is subsequently loaded with acetyl units using acetyl-CoA (Ac-CoA) on the cytoplasmic side of the membrane, as has been proposed by Babinger and co-workers (27). The Ac-HepG extracellular acetyl donor is then shuttled across the membrane by an unidentified process where it is utilized by LhaT (YpjA) to *N-*acetylate DA-LP substrates produced by Lsp-catalyzed proteolysis of pre-lipoproteins. Extracellular-facing HepG carrier can then be recycled to the cytoplasmic side and reloaded with acetyl donor for further rounds of lipoprotein *N-*acetylation. For clarity, only the C1 glyceryl mono-acetylated HepG donor is shown.

To begin to understand how these proteins are involved in lipoprotein *N*-acetylation, we generated recombinant PcrB, YvoF, and YpjA to reconstitute the putative activity *in vitro*. YpjA is an unannotated membrane protein with no known functional domains that is predicted to encode six transmembrane α-helical passes by DeepTMHMM (26). However, previous studies by Babinger and co-workers have shown that PcrB and YvoF enzymes work together to synthesize a low-abundance, archaea-type ether lipid of unknown function in *B. subtilis* (Fig. S5A) (27). PcrB condenses *sn*-glycerol 1-phosphate (G1P) with heptaprenyl pyrophosphate (HepPP) to form heptaprenylglyceryl phosphate (28, 29), which is subsequently dephosphorylated by a non-specific phosphatase prior to acetylation by YvoF to form acetylated heptaprenylglyceryl (Ac-HepG) with acetyl-coenzyme A (Ac-CoA) as donor (27). We hypothesized Ac-HepG could be the acetyl donor for lipoprotein modification, serving as a lipophilic shuttle that is loaded with acetyl units in the cytosol, transverses the membrane, and then is used by YpjA to *N*-acetylate the α-amino cysteine group of lipoproteins, which only becomes available after proteolysis by Lsp on the extracellular surface. We substituted the commercially available geranylgeranyl pyrophosphate (GGPP) (four isoprenyl repeats) for the native heptaprenyl pyrophosphate (seven isoprenyl repeats) carrier, as both PcrB and YvoF can accept the shorter substrate albeit with less efficiency (30). When using 100 µM Ac-CoA, all three potential acetyl donors for YpjA (monoacetyl Ac-HepG on C1 or C2 of the glyceryl unit along with Ac_2_-HepG) could be generated in *situ* (Fig. S5B). To this reaction mixture, recombinant YpjA from *B. subtilis* produced in *Escherichia coli* membranes lacking the endogenous lipoprotein *N*-acylation enzyme Lnt was added in tandem with a synthetic fluorescently labeled diacyl lipopeptide (DA-LP*) acceptor substrate. A faster migrating spot was observed by thin-layer chromatography (TLC) only under replete assay conditions that included Ac-CoA, YvoF, and membranes containing YpjA (Fig. 3A). In addition, the more hydrophobic lipopeptide product demonstrated time-dependent accumulation (Fig. 3B). Spots were scraped from plates, eluted, and analyzed by MALDI-TOF MS to determine the lipopeptide modification. Consistent with the addition of an acetyl group to form Ac-LP*, the Ac-CoA/YvoF/YpjA lipopeptide product was mass shifted by +42 Da (Fig. 3C and D). The genetics and *in vitro* biochemical reconstitution together support a new lipoprotein modification pathway that invokes a poly-isoprenoid carrier to supply acetyl donors to the extracellular surface for the integral membrane protein YpjA to transfer the ester-linked acetyl group from Ac-HepG to an amide-linked bond with the α-amino termini of DA-LP substrates (Fig. 4). We have thus annotated YpjA as LhaT (lipoprotein heptaprenylglyceryl *N*-acetyl transferase) in *B. subtilis* to reflect the enzymatic activity.

### The lipoprotein *N*-acetylation pathway has decayed in certain pathogenic *Bacillus* spp.

**Fig 5:**
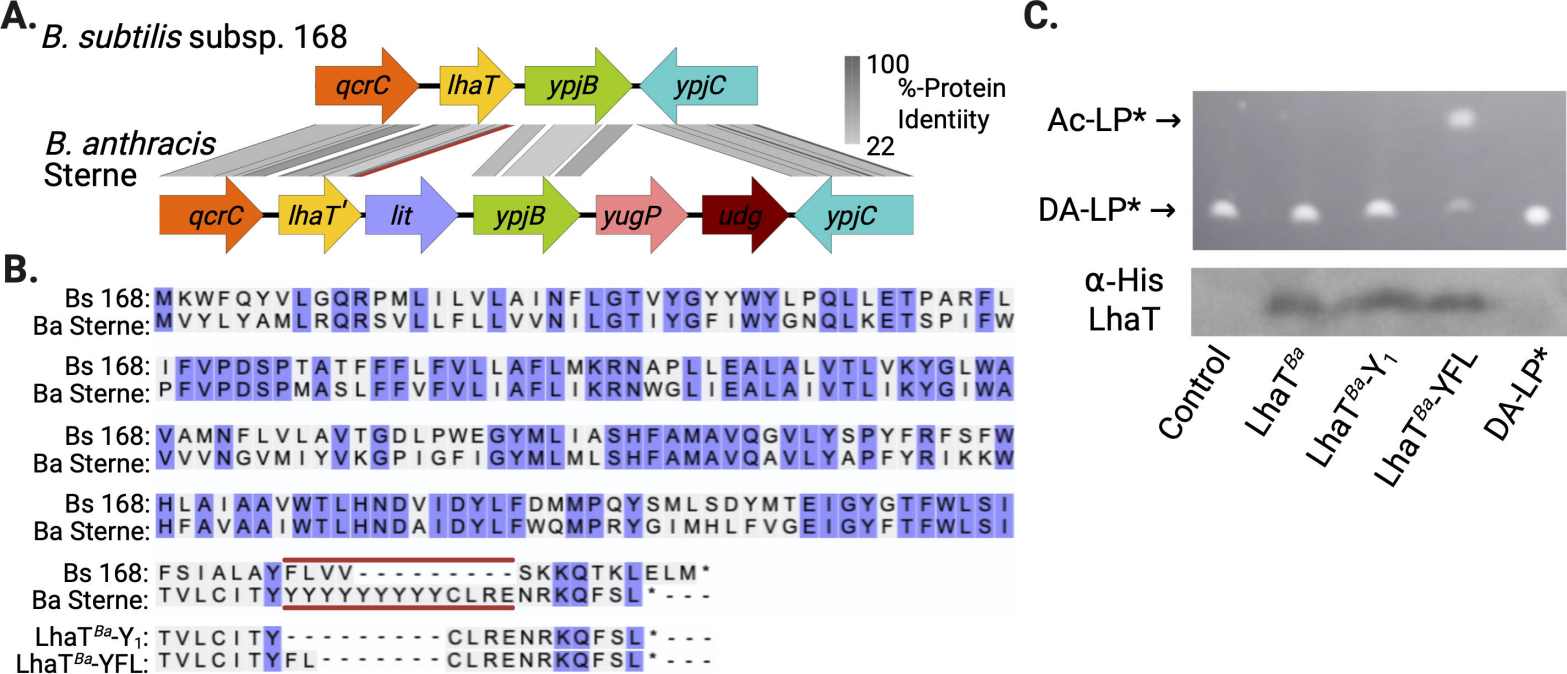
LhaT′ in *B. anthracis* is inactive due to a *C*-terminal poly-tyrosine insertion. (A) The *lhaT*(*ypjA*) gene loci from *B. subtilis* subsp. 168 (locus tags BSU_22540 to BSU_22510) and *B. anthracis* Sterne (locus tags AW20_1240 to AW20_1234) were aligned, and the percent protein sequence identity was calculated (gray shading) for shared genes using EasyFig 2.0 (31). The position of the 10-residue poly-tyrosine insertion is shown with the red bar. (B) Protein sequence alignment of LhaT from *B. subtilis* subsp. 168 (Bs168) and *B. anthracis* Strene (Ba Sterne) using UniProt (32). The poly-tyrosine insertion region (flanked by red lines) is 10-Tyr residues long in *B. anthracis* Sterne but ranges from 4 to 18 consecutive Tyr residues in various *B. anthracis* isolates (see Fig. S6A). The alignment of LhaT constructs with a single Tyr residue (LhaT*^Ba^*-Y_1_) or with a conserved α-helical transmembrane pass length corresponding to *B. subtilis* (LhaT*^Ba^*-YFL) is shown at the bottom. (C) Recombinant LhaT from wild-type *B. anthracis* Sterne (LhaT^Ba^), with a single Tyr residue (LhaT*^Ba^*-Y_1_), or with the corresponding *B. subtilis* sequence (LhaT*^Ba^*-YFL) was expressed in *E. coli* membranes, and the acetylation activity was measured using DA-LP* substrate as in Fig. 3A. Equivalent amounts of LhaT were normalized by immunoblotting for *N*-terminal His-tag (bottom panel). Control: empty *E. coli* membranes.

While *B. subtilis* strains make Ac-LP and are detected with high sensitivity by TLR2 (Fig. 1B), other *Bacillus* spp. such as *B. cereus* encode *lit* and make the TLR2 silent lyso-LP ligand (4, 13, 14). Curiously, however, apparent LhaT orthologs (>50% amino acid identity) still remain within the *B. cereus* subclade, including in genomes from *B. anthracis* (Fig. 5A). Local gene synteny is also generally conserved, except for the insertion of *lit* immediately downstream of *lhaT* in *B. anthracis* to form an operon with a shared upstream promoter. The co-expression of *lhaT-lit* on the same transcript would seem to disfavor possible lipoprotein chemotype switching by differential regulation of the two modification systems. Instead, close inspection of the LhaT C-terminus from *B. anthracis* suggests the protein may have lost function. A peculiar 10-residue long poly-tyrosine (poly-Tyr) run is present in *B. anthracis* Sterne (Fig. 5B). An equivalent poly-Tyr stretch, varying from 4 to 18 consecutive Tyr residues, is common among *B. anthracis* isolates of diverse origin (Fig. S6A). To test for function, recombinant *B. anthracis* Sterne LhaT was heterologously expressed in *E. coli* membranes and tested for activity as described above. The wild-type preparation containing *B. anthracis* (LhaT^Ba^) did not modify DA-LP* substrate (Fig. 5C). We then attempted to restore enzymatic activity by removing the entire poly-Tyr run except for a single Tyr residue (LhaT^Ba^-Y_1_). However, this construct also failed to yield any product. AlphaFold predicts the poly-Tyr run occurs at the membrane-water exit interface of the sixth α-helix transmembrane pass (Fig. S6B). While recombinant protein was expressed and incorporated into the membrane, we reasoned the LhaT^Ba^-Y_1_ construct may be too short to traverse the membrane and be rendered non-functional. We thus exchanged the poly-Tyr run with the equivalent three amino acid YFL sequence from *B. subtilis*. The resulting construct (LhaT^Ba^-YFL) readily synthesized *N*-acetylated Ac-LP* (Fig. 5C). Once more, heterologous ectopic expression of LhaT^Ba^ from a constitutive promoter in *B. subtilis* Δ*lhaT* failed to produce detectable Ac-LP by MALDI-TOF analysis (Fig. S7A). Expression of the *B. anthracis lhaT-lit* two-gene cassette with the native promoter in *B. subtilis* Δ*lhaT*, where an Ac-HepG donor pool is readily available, results in low TLR2 activation due to lyso-LP being the major chemotype (Fig. S7B). This indicates *B. anthracis* LhaT does not effectively compete with Lit for DA-LP substrate and supports pseudogene annotation (*lhaT’*) due to poly-Tyr insertion.

## DISCUSSION

Transposon library screening in *B. subtilis* revealed that at least three genes located at three distinct loci are strictly required for Ac-LP biosynthesis *in vivo* (Fig. 2), supporting the proposal of a new pathway for Ac-LP formation (Fig. 4). The *B. subtilis N*-acetylation pathway joins a growing list of genera and species-specific lipoprotein tailoring modifications distributed among Firmicutes. Unlike lyso-LP (20) and TA-LP (15, 33) biosynthesis, however, Ac-LP is uniquely unable to source the acyl donor from the membrane itself since glycerophospholipids typically lack two-carbon long acyl chains. We instead propose that the heptaprenylglyceryl carrier is loaded with cytoplasmic Ac-CoA and flipped across the membrane to supply LhaT (YpjA) for DA-LP *N*-acetylation. A polyprenylglyceryl-based carrier shuttle is a unique solution to the challenge of supplying extracellular high-energy acetyl donors. In other characterized extracellular acetylation pathways, a series of ping-pong transfers using juxtaposed amino acids to form transient acetyl-amino acid covalent intermediates shuttle acetyl units from cytosolic Ac-CoA/Ac-P*_i_* donors through the membrane interior (34, 35). Lipoprotein *N*-acetylation is mechanistically more akin to extracellular glycosylation pathways that also use lipophilic membrane polyprenyl carriers. Lipopolysaccharide O-antigens/glycosylations (36, 37), wall teichoic acids (38, 39), and peptidoglycan (40), among others, all use polyprenyl carriers pre-loaded on the cytoplasmic face with nucleoside diphosphate sugar substrates to transit high-energy glycosyl donors to the cell surface.

While the entire pathway could be reconstituted *in vitro* with just PcrB, YvoF, a non-specific phosphatase, and LhaT (YpjA) (Fig. 3), it is presently unclear how Ac-HepG/HepG transverses the membrane (Fig. 4). Bacterial glycosyl polyprenyl carriers are typically longer (C55, undecaprenyl with eleven isoprenoid units) and phosphorylated (41), requiring dedicated integral membrane flippase proteins for both the export of polyprenyl glycoconjugates (42, 43) as well as for the return of the polyprenyl phosphate to the cytosolic face (44–46). The small size, neutral charge, and higher hydrophobicity of Ac-HepG may circumvent the need for dedicated transporters, as crossing the membrane interior is not as energetically unfavorable in comparison to more hydrophilic carbohydrate-based cargo. Mass action alone could ensure directional cycling, with Ac-HepG being synthesized on the cytoplasmic face and consumed on the extracellular membrane face by LhaT. Likewise, HepG byproduct would be recycled back into the cytosol for reloading and subsequent rounds of acetylation using Ac-CoA. If the kinetics of concentration-driven membrane transversion alone are insufficient, it remains possible that the transporter was not identified in the transposon screen due to transporter redundancy. Alternatively, if a flippase that traffics other essential polyprenyl substrates is shared, it would not have been represented in our transposon library.

PcrB is part of a larger family of prenyl transferases that condense G1P with all *trans-*polyprenyl pyrophosphate substrates (29). The catalytic activity of PcrB was initially inferred from sequence similarity to archaeal geranylgeranylglyceryl phosphate synthases involved in isoprenoid membrane lipid biosynthesis (28, 47–50). The enzyme families have high sequence similarity and conserved G1P binding sites and share overall protein folds, with polyprenyl chain length discrimination conferred by the relative position of bulky side-chain residues protruding into the binding cavity (51). While the HepPP substrate is a common bacterial metabolite also used in menaquinone and sesquarterpenoid biosynthesis in *B. subtilis* (52), the cellular purpose of PcrB and its ultimate product Ac-HepG has been more enigmatic. It has been postulated that PcrB may have been originally acquired from archaea by horizontal gene transfer and then diverged in bacteria (28, 50, 53). A catalytic role as a transient carrier for acetyl groups, as opposed to being a structural membrane building block for an archeal-type lipid with ether-linked isoprenoid chains, is more consistent with the low overall abundance of HepG/Ac-HepG observed in *B. subtilis* membranes (28).

The lipoprotein heptaprenyl *N*-acetyl lipoprotein transferase LhaT (formerly YpjA) is predicted to encode six transmembrane α-helical passes harboring a single domain of unknown function (DUF1405). LhaT belongs to a branch of the recently described ATGY lipid metabolizing enzyme superfamily, which was recently defined in studies on the human integral membrane protein TMEM164 (54). Like LhaT, TMEM164 is also a bimolecular acyl transferase, moving the C20:4 *SN*_2_-acyl chain from phosphatidylcholine to ether-linked polyunsaturated fatty acid lyso-phospholipids. LhaT is thus far the only enzyme demonstrated to utilize Ac-HepG donor for acetylation transfer reactions. The proposed activity is further supported by genome co-occurrence analysis using the STRING protein-protein network database (55). PcrB/LhaT are among the highest scoring co-occurring genes, with a particularly wide distribution and tight association in genomes from certain bacterial (Firmicutes) and archaeal (Euryarchaeota) phyla. Archaeal lipoproteins are thought to be similar to bacterial counterparts in structure and function but contain poly-isoprenoid-modified glyceryl units that reflect the membrane lipid composition (56, 57). Interestingly, *N*-terminal lipoprotein acetylation in the haloalkaliphilic archaeon *Natronomonas pharaonis* has been reported (58), suggesting the bacterial Ac-LP pathway described here may operate in archaeal counterparts as well. The co-occurrence of PcrB with YpjA is not absolute, however, with PcrB enjoying a broader strain distribution than LhaT. Ac-HepG may therefore have cellular roles beyond just supplying acetyl units for lipoproteins. Many bacteria such as *Staphylococcus aureus*, *Listeria monocytogenes*, and *B. cereus*, known to make TA-LP (13, 15), DA-LP (13, 19), and lyso-LP (13, 14), respectively, do not synthesize Ac-LP yet encode PcrB/LhaT orthologs. Whether this is due to lipoprotein chemotype conversion and subsequent silencing of the Ac-LP pathway, as seen here in the *B. cereus* group, or if there is indeed a broader cellular function for Ac-HepG in other extracellular acetylation reactions is currently unknown.

**Fig 6:**
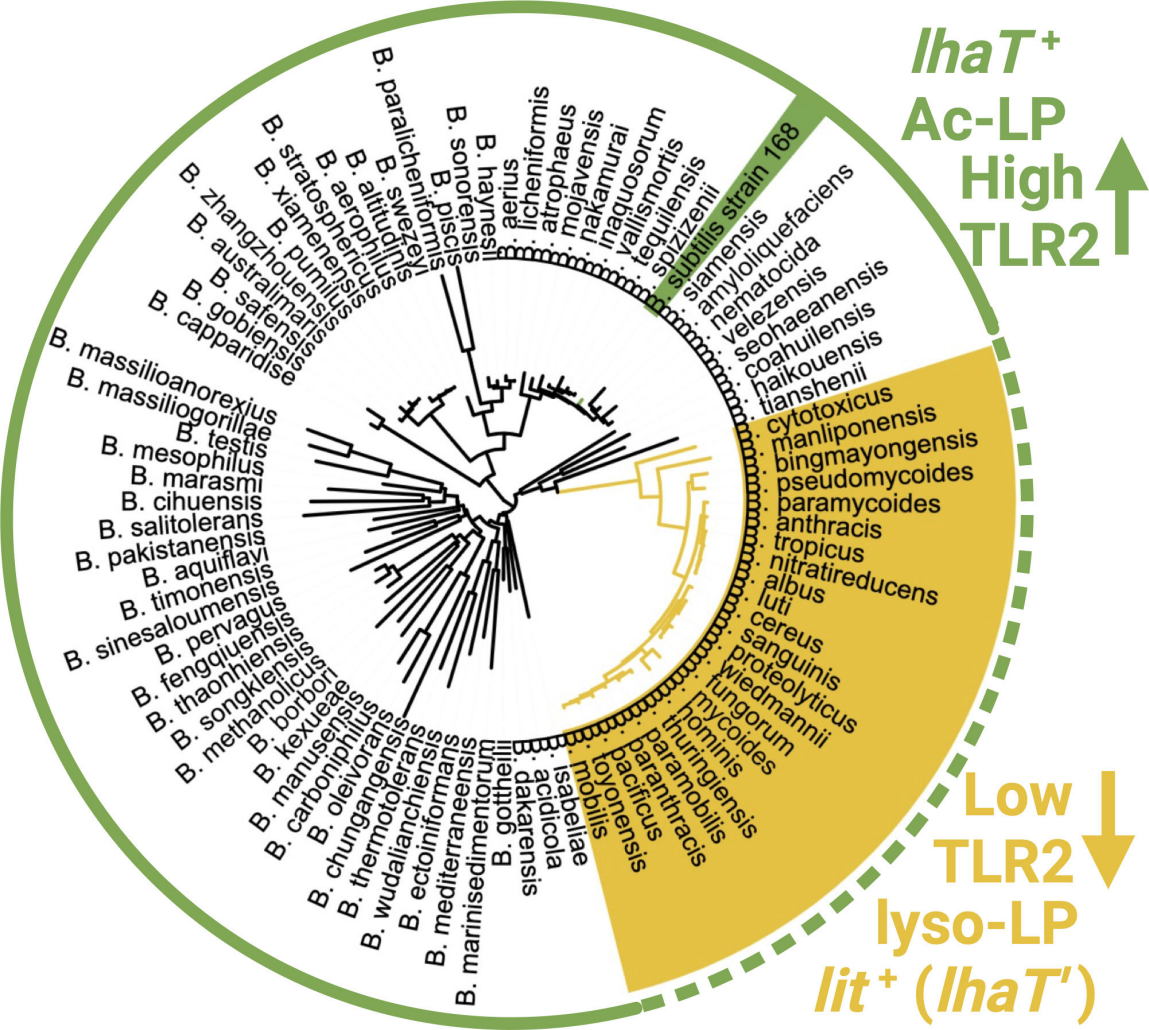
Phylogenetic analysis of LhaT and Lit distribution in *Bacillus* sp. A representative 16S rRNA sequence from each *Bacillus* sp. was used to construct a phylogenetic tree. While Lit sequence orthologs are narrowly distributed among the *B. cereus* group and make lyso-LP with low intrinsic TLR2-stimulating activity (mustard shading), LhaT orthologs are broadly distributed throughout the genus (green shaded line). Where LhaT is present and the Ac-LP pathway remains functional (LhaT^+^; i.e., *B. subtilis*), TLR2 (↑) activity is high (solid green line). To evade TLR2 (↓) orchestrated immunity (dashed green line), opportunist pathogenic lineages have converted to lyso-LP chemotypes through *lit^+^* acquisition and lipoprotein *N-*acetylation pathway decay (i.e., LhaT′ pseudogene, poly-Tyr insertion in *B. anthracis*).

All *Bacillus* spp. appear to encode genes for lipoprotein *N*-terminal modification (Fig. 6), suggesting there are general environment-dependent advantages to amidation. A potential reason for *N*-terminal acetylation/acylation comes from studies using select strains of *Listeria monocytogenes*, where copper ion has been shown to activate a two-component copper sensing (CopRS) signaling system and direct conversion of DA-LP to lyso-LP by transcriptional induction of a plasmid/transposon encoded *lit* gene (18–21). Given that copper co-induces both lipoprotein *N*-terminal modification and copper resistance genes, it has been proposed that copper binds DA-LP with higher affinity and inflicts more oxidative damage without *N*-terminal modification. There is a clear preference for which *N*-terminal modification among *Bacillus* spp., however. Out of the more than 180 classified taxa (59), Lit is confined to a narrow subclade within the *B. cereus* group housing the opportunistic food pathogen *B. cereus* and the causative agent of anthrax *B. anthracis* (Fig. 6). Phylogenomic analysis suggests that *lit* acquisition, Ac- to lyso-LP conversion, and hence suppression of TLR2-mediated detection are critical factors that differentiate environmental isolates from those with pathogenic potential. However, LhaT and other genes required for Ac-LP synthesis are uniformly distributed throughout the genera, requiring either tight regulation or pseudogenization of the endogenous Ac-LP synthesis pathway. In *B. anthracis* isolates, Ac-LP loss can be attributed to the insertion of a variable poly-Tyr run within the extreme *C*-terminal coding region of LhaT (Fig. 5; Fig. S6). In other *lit*^+^ species within the *B. cereus* group, loss of function mutations within LhaT orthologs are not as apparent. Whether these LhaT orthologs are also non-functional or if lesions have arisen in other steps involving Ac-HepG donor synthesis is currently unknown. Only pseudogenization of *lhaT* would retain an Ac-HepG donor pool for other putative extracellular acetyl transfer reactions. The Ac-LP pathway has likely decayed in other strains within this subclade besides *B. anthracis*, including *B. cereus*, where only lyso-LP has been detected (13, 14). In either case, synthesis of lyso-LP as opposed to Ac-LP suppresses TLR2 detection while still conferring the putative copper resistance associated with *N*-terminal lipoprotein modification.

## MATERIALS AND METHODS

### Bacterial strains and growth conditions

Strains of *Bacillus subtilis* were grown in lysogeny broth (lysogeny broth medium [LBM], 10 g tryptone, 10 g NaCl, 5 g yeast extract, and 3 mM MgSO4) supplemented with appropriate antibiotics (tetracycline, 3 µg/mL; kanamycin, 30 µg/mL; erythromycin, 5 µg/mL; and chloramphenicol, 10 µg/mL for plasmids and 5 µg/mL for chromosomal integration) at 37°C under constant aeration. Linearized DNA was directly transformed by natural competence into *B. subtilis*, while replicating plasmids were first passaged in *E. coli* TG1 to generate concatemeric DNA. Strain construction details, plasmids, and a list of strains used in this study can be found in the supplemental materials (Tables S1 and S2).

### Transposon library construction

The TnFLX transposon vector delivery shuttle plasmid pFK132 was used for transposon library construction in *B. subtilis* TXM1762 as described (22), except the *ermR* resistance marker was first exchanged with a *kanR* marker. The *kanR* marker was PCR amplified (primers TM2263-64 with pORF5 Tnp^+^ template) and integrated into *E. coli* stellar cells carrying both pFK132 and the Red recombinase expressing plasmid pKD46 (24). Cultures of *B. subtilis* TXM1762 with pTXM1665 were grown at room temperature with shaking for 16 h in LBM with chloramphenicol and kanamycin at 37°C, before dilution at 1:100 vol/vol into pre-warmed fresh media with no antibiotics and outgrown at 37°C for 6 h. Cultures of transposed *B. subtilis* TXM1762 were supplemented with glycerol (final 15% wt/vol) and stored as glycerol stocks at −80°C.

### TLR2 activation assay

HEK293 NF-κB-inducible SEAP reporter cells stably transfected with human TLR2/CD14 and endogenously expressing TLR1/6 (HEK-Blue hTLR2 or Hek-Blue-Lucia hTLR2; InvivoGen, San Diego, CA) were cultivated in 75 cm^2^ culture flasks in 15 mL Dulbecco’s modified Eagle medium (DMEM) supplemented with 10% fetal bovine serum , 2 mM L-glutamine, 50 U/mL penicillin, 50 µg/mL streptomycin, 100 µg/mL Normocin (InvivoGen), and 4 µL/mL HEK-Blue selection antibiotics at 37°C in 5% CO_2_. Aliquots of the transposon library glycerol stocks were diluted and plated to single colonies on LBM agar selection plates (cat 5 µg/mL) and incubated overnight at 37°C. Individual colonies were subcultured in 96-well microplates containing 200 µL of LBM and grown to stationary phase at 37°C for 18 h. Cultures were heat-killed by incubation at 58°C for 1 h and then diluted 900-fold vol/vol in phosphate-buffered saline (PBS). Extracts were thoroughly suspended before withdrawing 20 µL for HEK-Blue TLR2 SEAP reporter cell challenge in 96-well adherent microplates (~25,000 cells seeded per well in 180 µL of supplemented DMEM). Cells were incubated post-challenge for 20 h at 37°C in 5% CO_2_. Relative NF-κB-dependent SEAP secretion was quantified by measuring absorbance at *A*_620 nm_ using Quanti-Blue solution (InvivoGen) according to the manufacturer’s instructions. Approximately 6,000 transposon mutant colonies were screened in total (Fig. S2). Initial hits with TLR2 activity 10-fold lower than the control strain GKM1757 (*P*_pen_-*lit*) were repurified to single colonies and tested in replicates using CFU normalized cultures.

### Transposon insertion site mapping

Genomic DNA from validated transposon mutants with low TLR2-stimulating activity was isolated by guanidium thiocyanate-EDTA-sarkosyl extraction (60). DNA was digested with *Sau*3AI and ligated with T4 DNA ligase as described (22). Transposon insertion sites were determined by Sanger sequencing of inverse PCR products (primers TM2320-2321) using ligation reactions as template.

### Mass spectrometry

Lipoproteins were prepared for MALDI-TOF MS as previously described (61). In brief, bacteria grown to late exponential phase were treated with lysozyme and lysed with bead-beating. Lipoproteins were enriched with Triton-X-114 phase partitioning and separated on a 12% SDS-PAGE gel. Following transfer to a nitrocellulose membrane, bands were visualized with Ponceau S staining. Bands selected for MALDI-TOF MS analysis were trypsinized overnight and eluted from membranes. Single or multiple 0.5 µL layers of lipopeptide in 10 mg/mL α-cyano-4-hydroxycinnamic acid matrix were deposited onto a steel target plate. Mass spectra were collected using an Ultraflextreme (Bruker Daltonics) MALDI-TOF mass spectrometer.

### Recombinant protein expression

The PcrB expression vector pGKM1815 was built by amplifying the *pcrB* gene (primers TM2493-94) and cloning into pET22-N6X HIS-TEV that had been digested with *Nhe*I/*Hind*III. The plasmid was transformed into *E. coli* BL21(DE3) and grown in 1 L of LB with carbenicillin (100 µg/mL) to mid-log growth phase (OD_600 nm_ ~0.5) at 37°C. Cultures were shifted to 16°C, induced with 1 mM isopropyl β-D-1-thiogalactopyranoside (IPTG), and incubated with shaking for 16 h. Cells were collected by centrifugation (5,000 × *g*, 10 min), resuspended in 35 mL of column buffer (20 mM Tris, pH 8, 300 mM NaCl, 5 mM imidazole, 1 mM dithiothreitol, and 1 mM phenylmethylsulfonyl fluoride [PMSF]), and broken by three passes through a French pressure cell (14,000 lb/in^2^). Unbroken cells and debris were removed by centrifugation (18,000 × *g*, 20 min at 4°C). Recombinant *N*-terminal 6× His TEV site-PcrB was then purified from the ensuing supernatant using HisPur cobalt resin (Thermo Fisher Scientific) according to the manufacturer’s instructions. Protein was further purified using a HiLoad 26/60 Superdex 200 gel filtration column (GE Healthcare) pre-equilibrated with buffer (20 mM Tris-HCl, pH 8, 150 mM NaCl, and 10% glycerol). Fractions containing purified PcrB were pooled, concentrated, and supplemented with glycerol to a final 20% concentration before being flash frozen in liquid nitrogen and stored at −80°C.

The *yvoF* gene was amplified (primers TM2520-21) and cloned into pET22b(+) using the *Nhe*I/*Hind*III restriction sites to encode a 6× C-terminal His tag. The pAR1851 plasmid was transformed into *E. coli* BL21(DE3) and grown as described above for PcrB to induce protein expression. The cell pellet from 1 L of overnight culture was resuspended in 40 mL Solulyze Bacterial Protein Extraction Reagent (Gelantis) supplemented with 1 mM PMSF and incubated at room temperature for 10 min to lyse cells. The suspension was centrifuged at 18,000 × *g* for 15 min to pellet cell debris. A 4 mL aliquot of HisPur cobalt resin slurry (Thermo Fisher Scientific) was added to the supernatant containing soluble protein and incubated at 4°C for 1 h with gentle shaking. The suspension was collected by centrifugation (5,000 × *g*, 5 min), and the pelleted resin was washed once with 10 mL of wash buffer (0.5 mM *n*-dodecyl β-D-maltoside [DDM], 300 mM NaCl, 20 mM sodium phosphate, pH 7.4) containing 5 mM imidazole, and then followed by 5 × 1 mL washes with wash buffer containing 10 mM imidazole. For elution, 15× 1 mL washes were performed with the elution buffer (150 mM imidazole, 0.5 mM DDM, 300 mM NaCl, 20 mM sodium phosphate, pH 7.4). Individual washes were collected and screened for YvoF by SDS-PAGE analysis. Fractions were pooled, concentrated (Vivapsin, MWCO 10 kDa), and further purified using a pre-equlibrated (0.5 mM DDM, 20  mM Tris-HCl, pH 8, 300  mM NaCl, and 10% glycerol) HiLoad 26/60 Superdex 200 gel filtration column (GE Healthcare). Enriched fractions containing YvoF protein were pooled; glycerol was adjusted to 20% and flash frozen in liquid nitrogen for long-term storage at −80°C.

Expression vectors for the membrane protein YpjA (pGKM2519 for *B. subtilis* and pRW2552 for *B. anthracis*) were built to append an *N*-terminal 8× His tag with an in-frame 3 glycine-1 serine linker using *Nhe*I/*Hind*III digested pET22-N8 × 3GS and the corresponding PCR cassettes (primers RW3298-99 for *B. subtilis* YpjA and primer RW3376-77 for *B. anthracis* YpjA) assembled with InFusion cloning (Takara Biosciences). Constructs for *B. anthracis* YpjA encoding a single tyrosine residue (pRW2585) or a YFL motif (pTM2665) in place of the poly-Tyr run were made by inverse PCR from pRW2552 using primer pairs RW3406-06 and TM3447-48, respectively. All YpjA expression constructs were transformed into the *E. coli* BL21(DE3) derivative TXM2540, where the endogenous lipoprotein *N*-acylation Lnt gene has been deleted by overexpression of the lipoprotein Lol transport system and removal of the major lipoprotein Lpp, as previously described for *E. coli* K-12 (20). For each YpjA construct, 1 L of cells was grown and induced with 1 mM IPTG at 16°C as described above. Cell pellets were resuspended in 35 mL of Tris-buffered saline (20 mM Tris-HCl, pH 8, 150 mM NaCl, and 1 mM EDTA with 1 mM PMSF) and lysed using a French pressure cell (three passes at 14,000 lb/in^2^). After low-speed centrifugation (16,000 × *g* for 20 min at 4°C), the membranes were collected by ultracentrifugation at 110,000 × *g* for 1 h at 4°C. Membranes were thoroughly resuspended in 1.2 mL of buffer (20 mM Tris pH 8, 150 mM NaCl, and 20% glycerol), aliquoted, and frozen at −80°C until use in reconstitution assays.

### Reconstitution of the lipoprotein *N*-acetylation pathway

To generate Ac-HepG substrate with recombinant PcrB and YvoF, protocols described in Guldan et al. (28) and Linde et al. (27) were adapted as follows. Briefly, 4 µM of PcrB was incubated with 100 µM GGPP (Cayman Chemical) and 200 µM *sn*-glycerol-1 phosphate lithium salt (G1P, Sigma) in 5 mM MgCl_2_, 1 mM β-mercaptoethanol, 0.05% DDM (1 Plus Chem), 100 mM 2-[4-(2-hydroxyethyl)piperazin-1-yl]ethanesulfonic acid, pH 7.5 buffer in a total volume of 200 µL at 37°C overnight. To the overnight reaction, 10 units of Quick calf alkaline phosphatase (CIP, New England Biolabs) was added, incubated at 37°C for a further 2 h, and then inactivated by incubation at 80°C for 5 min. Next, YvoF (1.5 µM final) with various concentrations of acetyl-CoA (0–250 µM Ac-CoA, Cayman Chemical) was added, and the reaction continued at 37°C for 2 h. Products were extracted according to the method of Bligh and Dyer (BD) by adding 750 µL of 1:2 chloroform:methanol, 250 µL of chloroform, and 250 µL of water (62), with vortexing between each addition, before centrifugation at 1,000 × *g* for 5 min. The bottom chloroform layer was transferred to a clean Eppendorf tube and left in the chemical hood to evaporate. Products were resuspended in 10 µL of a single-phase BD mixture (chloroform:methanol:water, 52:105:42 vol/vol/vol), spotted on TLC Silica gel 60 plates (Sigma), developed in ethyl acetate/hexane (1:1 vol/vol), and visualized with iodine vapor staining.

To assay lipoprotein *N*-acetyl transferase activity, the Ac-HepG substrate reaction with 100 µM Ac-CoA was assembled as described above, except DDM was replaced with 0.04% lauryl maltose neopentyl glycol (Thermo Fisher Scientific) added concurrently with YvoF in a 45 µL total reaction volume. Prior to BD extraction, frozen aliquots of resuspended membranes (20 mM Tris, pH 8, 150 mM NaCl, and 20% glycerol) isolated from *E. coli* BL21(DE3) strain TXM2540 expressing *N*-terminally His-tagged YpjA with a 3× Gly-Ser linker from either *B. subtilis* or *B. anthracis*, YpjA variants, or vector-only control membranes were thawed and added together with 10 µM of the fluorescently labeled lipopeptide substrate peptide di-palmitoyl *S*-glyceryl-Cys-Ser-Ser-Gly-Lys-ε-dansyl (DA-LP*, Biosynth). Reactions were incubated overnight at 37°C. To extract the products, an equal volume of 70% ethanol (50 µL) was added, followed by the addition of 30 µL of chloroform. After vortexing, tubes were centrifuged (2 min at 13,000 × *g*); the lower chloroform phase was transferred to a clean tube and evaporated in the chemical hood until the volume decreased to about ~5 µL. Reactions were analyzed by spotting on Silica gel 60 TLC plates (Sigma), developed in chloroform:methanol:water:acetic acid (65:25:4:1 vol/vol/vol/vol), and visualized by UV light excitation (365 nm). For direct comparison of YpjA specific activities, recombinant protein levels were normalized by immunoblotting onto polyvinylidene difluoride membranes blocked with 5% non-fat dry milk. Membranes were then washed with 6× His-tag monoclonal mouse antibody (Proteintech) and horseradish peroxidase-conjugated goat antimouse secondary antibody (Biotium) that had both been diluted 1:5,000 vol/vol in PBS with 0.05% Tween detergent and 2% non-fat dry milk.

## SUPPLEMENTAL MATERIAL

Supplemental material mbio.00996-25-s0001.pdf Supplemental tables, figures, and experimental details.

ASM does not own the copyrights to Supplemental Material that may be linked to, or accessed through, an article. The authors have granted ASM a non-exclusive, world-wide license to publish the Supplemental Material files. Please contact the corresponding author directly for reuse.
