# Supplementary material for: Lipoprotein N-terminal modification in Bacillus: a new paradigm for extracellular acetylation and species-dependent Toll-like receptor 2 immunomodulation
Source: mBio. 2025 Jul 8;16(8):e00996-25. doi: 10.1128/mbio.00996-25 (PMC12345273; doi:10.1128/mbio.00996-25)
Supplement: Supplemental material — Supplemental tables, figures, and experimental details. [file mbio.00996-25-s0001.pdf]

# SUPPLEMENTARY MATERIALS

## Lipoprotein *N*-terminal modification in *Bacillus*: A new paradigm for extracellular acetylation and species-dependent Toll-like receptor 2 immunomodulation

Gloria Komazin[a], Rachel M. Wigmore[a], Aditi M. Ranade[b], Amena A. Rizk[a], John H. Gardiner IV[b], and Timothy C. Meredith[a,b]*

[a] Department of Biochemistry and Molecular Biology, The Pennsylvania State University, University Park, Pennsylvania, USA
[b] The Huck Institutes of the Life Sciences, The Pennsylvania State University, University Park, Pennsylvania, USA

* Address correspondence to Timothy C. Meredith (txm50@psu.edu).

**This PDF file includes:**

- **Supplementary Methods**
- **Supplementary Tables S1 and S2**
- **Supplementary Figures S1 to S7**

## Supplementary Methods

**Strain and plasmid construction.** The *hag* gene encoding flagellin was deleted by assembling PCR fragments corresponding to the flanking DNA homology arms (primers TM2366-67 and TM2368-69, all primers are listed in Supplementary Table S2) with an intervening tetracycline resistance cassette (primers TM51-52) in pBR322 (primers TM2352-53) using In-Fusion Snap assembly (Takara Biosciences). The resulting plasmid (pTXM1753) was linearized by restriction digest and transformed into *B. subtilis* by natural competence using a protocol adapted from Burby and Simmons (1). In brief, a 2 mL culture of LM (LB supplemented with 3 mM $MgSO_4$) was inoculated 1:100 v/v with an overnight culture and grown at 37°C to an $OD_{600}$ of between 1.0 and 1.5. A 20 mL aliquot of the LM culture was transferred to 500 mL prewarmed filter-sterile competence (MD) medium (1x PC buffer [10x PC buffer is 107 g/L $K_2HPO_4$, 60 g/L $KH_2PO_4$, 10 g/liter trisodium citrate·$(H_2O)_5$], 2% glucose, 50 µg/mL tryptophan, 50 µg/mL phenylalanine, 11 mg/ml ferric ammonium citrate, 2.5 mg/mL potassium aspartate, 3 mM $MgSO_4$ ]) and incubated for 4 hrs. Linearized plasmid was added and the culture incubated for another 1.5 hrs before plating on relevant antibiotic LB agar. Gene replacement was confirmed by PCR (primers TM2370-71) and sequencing. Targeted gene deletions (*gene::*FRT) were likewise constructed by assembly of PCR fragments of the upstream homology arm (primers P1 and FRT P2 KO), the downstream homology arm primers (primers FRT P3 KO and P4 KO), and the kanamycin resistance (*kanR*) cassette [primers P1 kan FRT for and P2 kan FRT rev from pORF5 $Tnp^+$ template (2)] with the pBR322 plasmids backbone (primers TM2352-53). Verified plasmids were linearized by restriction digest, transformed into *B. subtilis*, and gene replacement with *kanR* flanked by FRT recombination sites confirmed by PCR analysis using up/downcheck primers. The *kanR* marker was removed using the FRT recombinase flippase expressing plasmid pTXM1831, which was subsequently cured by passaging at nonpermissive temperature (37°C). Gene deletion mutants were back complemented by integration within *lacA* using the vector pAX01 (3). Vectors for YvoF or YpjA under control of the $P_{pen}$ promoter were built by amplifying the respective genes

(primers TM2505-06 and RW2885-2642) and inserting into the inverse PCR product of pTXM1640 (primers TM2581-82) to make plasmids pTXM1920 and pRW2136, respectively. The PcrB complementation vector was built by PCR amplification of *pcrB* (primers TM2507-08) with its native promoter ($P_{pcrB}$) and inserted into *EcoR*I/*Asc*I digested pGKM1754. Back complementation of *ypjA* by chromosomal integration within the *amyE* locus was achieved using the plasmid pDL (4). The $P_{pen}$-*ypjA* from *B. subtilis*-terminator cassette was amplified (primers RW2856-57) from pRW2136 and joined with *Sac*I/*BamH*I digested pDL vector to make pRW2139. This construct was then amplified by inverse PCR (primers TM2581-82) and ligated with *B. anthracis ypjA* (primers RM3344-45) to complete pRW2532.

Strains of *B. subtilis* expressing the *B. cereus lit* gene were constructed by chromosomal integration within *lacA* using the vector pAX01 (3). Plasmid pTXM1640 was assembled from PCR fragments encoding *lit* (BC_1525, primers TM2145-46 with *B. cereus* genomic DNA template), the $P_{pen}$ promoter (primers TM2249-2139 with pTM402 template), and the *blaZ* transcriptional terminator (primers TM2582-2248 with pCN59 template) with *Sac*I/*Sac*II digested pAX01 plasmid backbone. Sequence verified plasmids linearized by restriction digest and transformed into *B. subtilis* as above. To introduce the $P_{nat}$ promoter of *lit* in *B. cereus* in pGKM1754, pTXM1640 was amplified by inverse PCR (primers TM2373-76) and joined with the 188-bp putative promoter region located upstream of BC_1525 (primers TM2374-75) using In-Fusion Snap assembly (Takara Biosciences).

### Table S1: Bacterial strains and plasmids

| Strains and plasmids | Relevant Genotype/Phenotype[1] | Source or reference |
|---|---|---|
| KA428 | Wildtype *B. subtilis* subsp. *subtilis* 168, Ac-LP | Lab Stock |
| GKM1755 | KA428 *hag::tetM*, flagella negative, Ac-LP; $Tet^r$ | This study |
| GKM1757 | GKM1755 *lacA::ermR* $P_{pen}$-*lit* from *B. cereus*, Ac-/lyso-LP mixture; $Tet^r$ $Erm^r$ | This study |
| TXM1762 | GKM1755 *lacA::ermR* $P_{nat}$-*lit* from *B. cereus*, Ac-/lyso-LP mixture; $Tet^r$ $Erm^r$ | This study |
| GKM1792 | TXM1762 *yvoD::tn* (TnP4E6); $Tet^r$ $Erm^r$ $Cat^r$ | This study |
| GKM1796 | TXM1762 *yvoF::tn* (TnP13C9); $Tet^r$ $Erm^r$ $Cat^r$ | This study |
| GKM1797 | TXM1762 *yvoE::tn* (TnP20D2); $Tet^r$ $Erm^r$ $Cat^r$ | This study |
| GKM1799 | TXM1762 *pcrB::tn* (TnP35E5); $Tet^r$ $Erm^r$ $Cat^r$ | This study |
| GKM1890 | TXM1762 *yvoE::tn* (TnP15H11); $Tet^r$ $Erm^r$ $Cat^r$ | This study |
| GKM1891 | TXM1762 *ypjA::tn* (TnP16D10); $Tet^r$ $Erm^r$ $Cat^r$ | This study |
| GKM1830 | TXM1762 *lgt::tn* (TnP2B6); $Tet^r$ $Erm^r$ $Cat^r$ | This study |
| TXM1795 | GKM1792 *lacA::kanR*; $Tet^r$ $Cat^r$ $Kan^r$ $Erm^s$ | This study |
| GKM1802 | GKM1796 *lacA::kanR*; $Tet^r$ $Cat^r$ $Kan^r$ $Erm^s$ | This study |
| GKM1803 | GKM1797 *lacA::kanR*; $Tet^r$ $Cat^r$ $Kan^r$ $Erm^s$ | This study |
| GKM1807 | GKM1799 *lacA::kanR*; $Tet^r$ $Cat^r$ $Kan^r$ $Erm^s$ | This study |
| TXM1836 | GKM1830 *lacA::kanR*; $Tet^r$ $Cat^r$ $Kan^r$ $Erm^s$ | This study |
| TXM1960 | GKM1890 *lacA::kanR*; $Tet^r$ $Cat^r$ $Kan^r$ $Erm^s$ | This study |
| TXM1961 | GKM1891 *lacA::kanR*; $Tet^r$ $Cat^r$ $Kan^r$ $Erm^s$ | This study |
| TXM1915 | TXM1762 *pcrB::*FRT; $Tet^r$ $Erm^r$ | This study |
| TXM1916 | TXM1762 *yvoE::*FRT; $Tet^r$ $Erm^r$ | This study |
| TXM1917 | TXM1762 *yvoF::*FRT; $Tet^r$ $Erm^r$ | This study |
| TXM1918 | TXM1762 *yvoD::*FRT; $Tet^r$ $Erm^r$ | This study |
| TXM1939 | TXM1762 *ypjA::*FRT; $Tet^r$ $Erm^r$ | This study |
| GKM1832 | KA482 *yvoD::*FRT | This study |
| GKM1833 | KA482 *yvoE::*FRT | This study |
| GKM1834 | KA482 *yvoF::*FRT | This study |
| GKM1835 | KA482 *pcrB::*FRT | This study |
| TXM1938 | KA428 *ypjA::*FRT | This study |
| TXM1925 | GKM1834 *lacA::*$P_{pen}$-*yvoF*; $Erm^r$ | This study |
| GKM1843 | GKM1835 *lacA::*$P_{pcrB}$-*pcrB*; $Erm^r$ | This study |
| RW2143 | TXM1938 *lacA::*$P_{pen}$-*ypjA*; $Erm^r$ | This study |
| RW2205 | TXM1939 *amyE::*$P_{pen}$ -*ypjA* from *B. subtilis;* $Cat^r$ | This study |
| RW2535 | TXM1939 *amyE::*$P_{pen}$ -*ypjA* from *B. anthracis* Sterne; $Cat^r$ | This study |
| RW2625 | TXM1938 *amyE::*$P_{pen}$-*ypjA* from *B. anthracis* Sterne; $Cat^r$ | This study |
| RW2673 | TXM1938 *lacA::*$P_{nat}$-*ypjA-lit* from *B. anthracis* Sterne; $Cat^r$ | This study |
| KA477 | *Bacillus cereus* ATCC14579 | ATCC |
| RW2522 | *B. anthracis* strain Sterne | Lab Stock |
| *E. coli* TG1 | F' *traD36 proAB lacIqZ* ΔM15] *supE thi-1* Δ(*lac-proAB*) Δ(*mcrB-hsdSM*)5(rK - mK -); *recA*$^+$ | (Lucigen) |
| TXM2540 | *E. coli* BL21(DE3) *lpp::catR* (pTXM1026) *lnt::specR by* P1*vir* transduction; $Kan^r$ $Cat^r$ $Spec^r$ | This study |
| JT409 | *Staphylococcus aureus* SA113 *agr::tetM* | Ref. (5) |
| | | |

| **Plasmids** | | |
|---|---|---|
| pAX01 | pBR322- ori *lacA:: xylR* $P_{xylA}$ MCS *ermR* vector; $Carb^r$ | Ref. (3) |
| pTXM1753 | pBR322- *hag::tetM*; $Carb^r$ | This study |
| pGKM1754 | pAX01- *lacA::ermR* $P_{nat}$-*lit blaZ* TT from *B. cereus*; $Carb^r$ | This study |
| pTXM1640 | pAX01- *lacA::ermR* $P_{pen}$-*lit blaZ* TT from *B. cereus*; $Carb^r$ | This study |
| pTM402 | pT181- *repC*- cop 623 ori sso; 1-kb DNA Φ 11 fragment; mini-Tn and outward facing $P_{pen}$ promoter | Ref. (6) |
| pCN59 | *blaZ* transcriptional terminator source | Ref. (7) |
| pFK132 | $P_{sigB}$ HMAR transposase with Mariner Tn::*catR* cassette *ermR*; $ori^{TS}$*Bs* $spec^R$ shuttle vector; $Cat^r$ $Erm^r$ $Spec^r$ | Ref. (8) |
| pTXM1665 | pFK132 *ermR::kanR*; $Cat^r$ $Kan^r$ $Spec^r$ | This study |
| pORF5 $Tnp^+$ | *kanR* cassette source; $Kan^r$ | Ref. (2) |
| pGKM1809 | pAX01- *pcrB::kanR-FRT* deletion vector; $Carb^r$ | This study |
| pGKM1810 | pAX01- *yvoE::kanR-FRT* deletion vector; $Carb^r$ | This study |
| pGKM1811 | pAX01- *yvoF::kanR-FRT* deletion vector; $Carb^r$ | This study |
| pGKM1812 | pAX01- *yvoD::kanR-FRT* deletion vector; $Carb^r$ | This study |
| pGKM1922 | pAX01- *ypjA::kanR-FRT* deletion vector; $Carb^r$ | This study |
| pTXM1831 | pKFC- $ori^{TS}$*Bs* $P_{pen}$ FRT recombinase flippase blaZ TT shuttle vector; $Carb^r$ $Cat^r$ | Lab stock |
| pTXM1920 | pAX01- *ermR* $P_{pen}$ -*yvoF blaZ* TT; $Carb^r$ | This study |
| pGKM1822 | pAX01- *ermR* $P_{pcrB}$ -*pcrB blaZ* TT; $Carb^r$ | This study |
| pRW2136 | pAX01- *ermR* $P_{pen}$ -*ypjA blaZ* TT from *B. subtilis*; $Carb^r$ | This study |
| pTXM1026 | pBBR1 *ori* KanR-*lolCDE* (expression driven by $P_{kan}$) with PA3286; $Kan^r$ | Ref. (9) |
| pET22-N6X HIS-TEV | pET22b(+)- *N*-terminal 6X His tag TEV protease with *Nhe*I/*Hind*III cloning site | Lab stock |
| pET22b(+) | pET22b(+)- *C*-terminal 6X His tag with *Nde*I/*Hind*III cloning site | Novagen |
| pET22-N8X 3GS | pET22b(+)- *N*-terminal 8X His tag-3Gly-1Ser linker *Nhe*I/*Hind*III cloning site | Lab stock |
| pGKM1815 | pET22b(+)- *N*-terminal 6X His TEV protease site PcrB | This study |
| pAR1851 | pET22b(+)- *C*-terminal 6X His YvoF | This study |
| pGKM2519 | pET22b(+)- *N*-terminal 8X His-3Gly-1Ser linker *B. subtilis* YpjA | This study |
| pRW2552 | pET22b(+)- *N*-terminal 8X His-3Gly-1Ser linker *B. anthracis* YpjA | This study |
| pRW2585 | pET22b(+)- *N*-terminal 8X His-3Gly-1Ser linker *B. anthracis* Y1 YpjA | This study |
| pTM2665 | pET22b(+)- *N*-terminal 8X His-3Gly-1Ser linker *B. anthracis* YpjA YFL substitution | This study |
| pDL | *amyE::*MCS *bgaB catR* cloning vector; $Carb^r$ | Ref. (4) |
| pRW2139 | pDL *amyE::*$P_{pen}$- *B. subtilis ypjA catR*; $Carb^r$ | This study |
| pRW2532 | pDL *amyE::*$P_{pen}$- *B. anthracis* Sterne *ypjA catR*; $Carb^r$ | This study |
| pRW2593 | pax01 *lac::B. anthracis* Sterne $P_{nat}$-*ypjA-lit catR;* $Carb^r$ | This study |

[1] Tn- transposon; TT- transcriptional terminator; TS- temperature sensitive; FRT- flipase recombination target; $Antibiotic^r$- resistant; $Antibiotic^s$- sensitive; Erm- erythromycin; Kan-kanamycin; Cat- chloramphenicol; Spec- spectinomycin; Carb- carbenicillin; $Tet^r$- tetracycline.

**Table S2: Primers used in this study**

| Primer Name | Primer Sequence |
|---|---|
| TM2352- pBR322 for | AGCTTGCGGCCGCGTCATCATTCTTGAAGACG |
| TM2353- pBR322 rev | TAAAGGCATCAAATAATCCTGATATTGTCTG |
| TM2366- hag v2 P1 | ACGCGGCCGCAAGCTTAGGCTGAGCCGCATAAGC |
| TM2367- hag v2 P2 | TGGGAATTCTAATCGAGCGCTGCAATATTGTGG |
| TM2368- hag v2 P3 | TGCAGGCCTTATCACAGCCGCAAAACGTACTTC |
| TM2369- hag v2 P4 | TATTTGATGCCTTTACAAATTGTAATCGCCTTG |
| TM51- TetM for *Stu*I | GTGATAAGGCCTGCAACCCAAATCTCGCAATTTG |
| TM52- TetM rev *EcoR*I | CGATTAGAATTCCCATATTTATATAACAAC |
| TM2370- hag v2 upcheck | GATGTGCAGATTACGCCGCGAAAGC |
| TM2371- hag v2 downcheck | GTTCACTGTTTTTGCTGTTGTTTCG |
| Tm2249- $P_{pen}$ for *Sac*I pAX01 | AAGAACGTCCCGGGGAGCTCGCATGCAAGCTAATTC |
| TM2139- $P_{pen}$ rev | TCTAGAGTCGACAATATTTGATTG |
| TM2145- Bc *lit Xba*I for | TATTGTCGACTCTAGACTTTATGATGGTTAGAAC |
| TM2146- Bc *lit Asc*I rev | TTTAGAATAGGCGCGCCCGTTTTACAAAGAAAAAAATTC |
| TM2582- *blaZ* TT *Asc*I for | CGCGCCTATTCTAAATGCATAATAAATAC |
| TM2248- *blaZ* TT *Sac*II | AAGAGTGCGGCCGCCCGCGGCGCCTGTCACTTTGC |
| TM2373- pAX01 $P_{nat}$ rev | CTTTATGATGGTTAGAACATTTAGG |
| TM2376- pAX01 $P_{nat}$ for | GGACGTTCTTGCCATGGAATTAGCTTGCATG |
| TM2374- $P_{nat}$ *lit* for | ATGGCAAGAACGTCCCGGAATTCGTTGCAAAG |
| TM2375- $P_{nat}$ *lit* rev | CTAACCATCATAAAGCCGAGCGTTGTCTTAACAT |
| TM2250- *lacA* Bs upcheck | GAATTTTGTGTCCGGATTAACCGCAG |
| TM2251- *lacA* Bs downcheck | GAATCCGCCCATATCGAGCGGAGC |
| TM2263- P1 kanR into Tnflx | GCAAAATTCATATAACCAAATTAAAGAGGGTTATAATGAACGA GAGCGAACCATTTGAGG |
| TM2264- P2 kanR into Tnflx | GGCACACGAAAAACAAGTTAAGGGATGCAGTTTATGCATCCC TTGGTACTAAAACAATTC |
| TM2320- Tnflx2 for | CGCCCTATAGTGAGTCGTGG |
| TM2321- Tnflx2 rev | CGAATTGGAGCTCGCGAGCC |
| TM2481- pcrB P1 KO | ACGCGGCCGCAAGCTCTGGTCAATCCTGCT |
| TM2482- pcrB FRT P2 KO | ATAGGAACTTCGCACGTCATACGTTCCTC |
| TM2483- pcrB FRT P3 KO | AGTATAGGAACTTCAAAACAGTAGCGGCTGTG |
| TM2484- pcrB P4 KO | TATTTGATGCCTTTAATCTTGATGCCTTCATC |
| TM2488- yvoD P1 KO | ACGCGGCCGCAAGCTCCGAAATCTTGCAGTCATC |
| TM2487- yvoD FRT P2 KO | ATAGGAACTTCGCATCCCTCATCCCCCTTTGTTGACGTCTACT C |
| TM2472- yvoD FRT P3 KO | AGTATAGGAACTTCAGCGGTTGGAAAGGAAGCAATATGA |
| TM2432- yvoD P4 KO | TATTTGATGCCTTTACGTGAATCAAATACTC |
| TM2475- yvoE P1 KO | ACGCGGCCGCAAGCTGGCAGCCATCATCTTC |
| TM2473- yvoE FRT P2 KO | ATAGGAACTTCGACTCATATTGCTTCCTTTCC |
| TM2474- yvoE FRT P3 KO | AGTATAGGAACTTCACAAATCGTTGGAGTGAAG |
| TM2477- yvoE P4 KO | TATTTGATGCCTTTACAGAAAACCAAGACCCTG |
| TM2437- yvoF P1 KO | ACGCGGCCGCAAGCTCATCCAGTTTTTAAGAG |
| TM2478- yvoF FRT P2 KO | ATAGGAACTTCGCACAGATTACTTCACTC |

| | |
|---|---|
| TM2480- yvoF FRT P3 KO | AGTATAGGAACTTCATTGAAAAAGTCCGCTG |
| TM2440- yvoF P4 KO | TATTTGATGCCTTTACTCAATGATCCGTATG |
| TM2600- ypjA P1 KO | ACGCGGCCGCAAGCTCAGGGGATTTCAGGAC |
| TM2601- ypjA P2 FRT KO | ATAGGAACTTCGCATCGGCCGCTGCCCTAATAC |
| TM2602- ypjA P3 FRT KO | AGTATAGGAACTTCACAAACGAAGCTTGAGCTGATGTAA |
| TM2603- ypjA P4 KO | TATTTGATGCCTTTAGAAGGATTTACGCTTGATG |
| TM2479- P1 kan FRT for pcrB/yvoF | GTGCGAAGTTCCTATTCTCTAGAAAGTATAGGAACTTCGGTAA GATTATACCGAG |
| TM2469- P1 kan FRT for yvoD/ypjA | ATGCGAAGTTCCTATTCTCTAGAAAGTATAGGAACTTCGGTAA GATTATACCGAG |
| TM2476- P1 kan FRT for yvoE | AGTCGAAGTTCCTATTCTCTAGAAAGTATAGGAACTTCGGTAA GATTATACCGAG |
| TM2470- P2 kan FRT rev pcrB/yvoD/yvoE/yvoF/ypjA | TGAAGTTCCTATACTTTCTAGAGAATAGGAACTTCGATGCAGT TTATGCATCCC |
| TM2485- pcrB upcheck | CTCCTTGTGCCAGCTTTAGCTC |
| TM2486- pcrB downcheck | GTCCTTCACCGAACTCATTATCAC |
| TM2441- yvoF downcheck | GAAATTACGTACAGCGAACACAGCG |
| TM2604- ypjA upcheck | GGCTGCACATTTTATTATGATCCG |
| TM2605- ypjA downcheck | CGATGTTCACGATGTGCTCGGAG |
| TM2581- $P_{pen}$ rev *Sal*I | CTAGCGAACCTTTGCGAGTCGACAATATTTG |
| TM2582- TT for *Asc*I | CGCGCCTATTCTAAATGCATAATAAATAC |
| TM2505- pAX01 $P_{pen}$ yvoF for | GCAAAGGTTCGCTAGACAAATCGTTGGAGTG |
| TM2506- pAX01 $P_{pen}$ yvoF rev AscI | TTTAGAATAGGCGCGCAAAAAAAGTCCGCTGATG |
| RW2885- pAX01 $P_{pen}$ ypjA Bs for | GCAAAGGTTCGCTAGATATTATTTTTAC |
| RW2642- pAX01 $P_{pen}$ ypjA Bs rev *Asc*I | TTTAGAATAGGCGCGAGGGACAAGGATAGACCAAC |
| TM2507-pAX01 $P_{pcrB}$ pcrB for *EcoRI* | GAACGTCCCGGAATTCGGTTTCCATCTCTCAGC |
| TM2508- pAX01 $P_{pcrB}$ pcrB rev *Asc*I | TTTAGAATAGGCGCGCATAAACCAAATCCGTAC |
| TM2493- pcrB pET22 for *Nhe*I | CTTCCAATCCGCTAGCTACGATGTAACGGAGTG |
| TM2494- pcrB pET22 rev *Hind*III | TTAGCAGCCCAAGCTTACTCGCCTTTCACAGC |
| TM2520- yvoF pET22 for *Nde*I | AAGGAGATATACATATGAGAAAAACAGATCGTCATC |
| TM2521- yvoF pET22 C-term 6XHis rev *Xho*I | GGTGGTGGTGCTCGAGGGCTTCAGCGGACTTTTTC |
| RW3298 - Bs YpjA pET22 for *Nhe*I | CCAATCCGCTAGCATGAAATGGTTTC |
| RW3299 - Bs YpjA pET22 rev *Hind*III | GCAGCCCAAGCTTACATCAGCTC |
| RW3376- Ba YpjA pET22 for *Nhe*I | GTGGGAGTGCTAGCATGGTTTATTTGTATG |
| RW3377- Ba YpjA pET22 rev *Hind*III | CAGCCCAAGCTTATAAAGAAAATTG |
| RW3405- Ba $Y_1$ YpjA pET22 for | ATTACATATTGCTTACGTGAGAACC |
| RW3406- Ba $Y_1$ YpjA pET22 rev | ACGTAAGCAATATGTAATACATAGC |
| TM3447- Ba YFL YpjA pET22 for | CATATTTTCTTTGCTTACGTGAGAACCG |
| TM3448- Ba YFL YpjA pET22 rev | AGCAAAGAAAATATGTAATACATAGC |
| RW2856- pAX01 up promoter | ATTCCCGGGGAGCTCGCATG |
| RW2857- pAX01 down term | CCAGTGCAGGTTGCTTGATATATG |
| RW3344- Ba pDL YpjA for | GCAAAGGTTCGCTAGAGGTGTTGGAC |
| RW3345- Ba pDL YpjA rev | TTTAGAATAGGCGCGACATATTCTAAC |
| RW3359- Ba pnat-ypjA-lit for | ATGGCAAGAACGTCCAGCTGAAGAAG |
| RW3360- Ba pnat-ypjA-lit rev | TTTAGAATAGGCGCGACAAAAAAATTC |

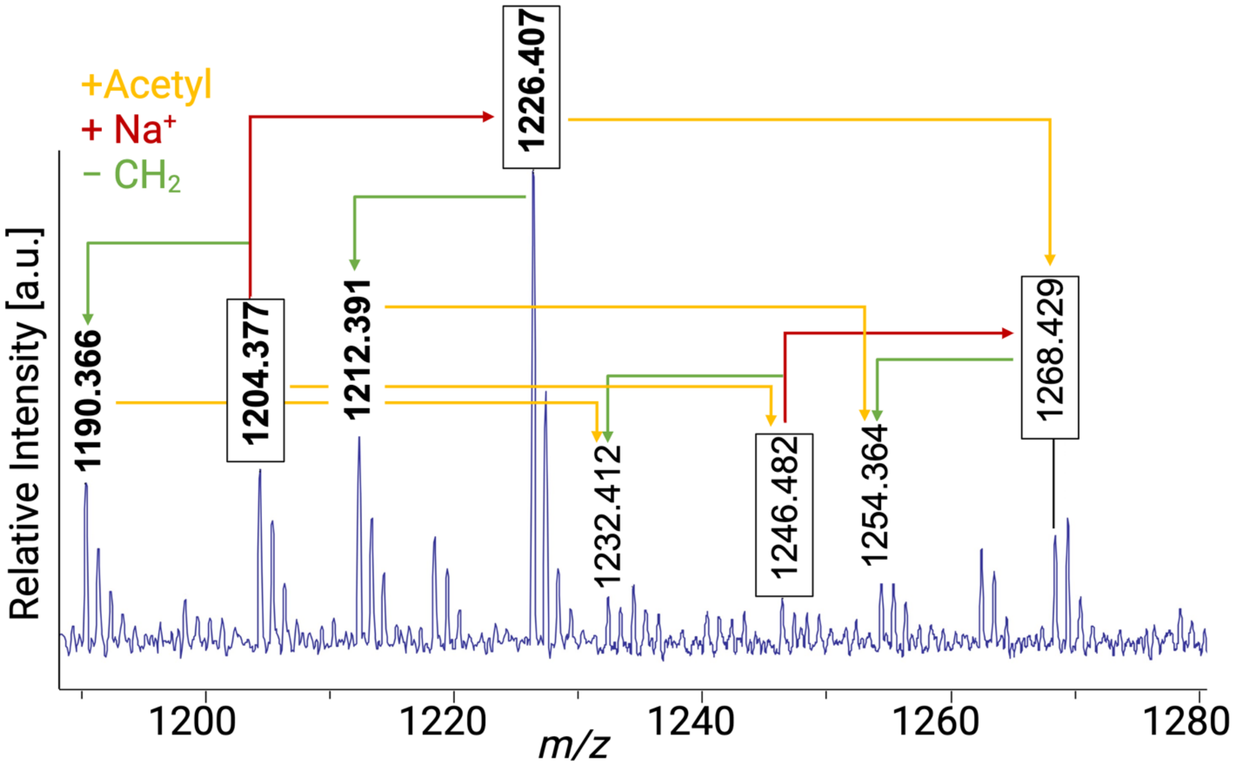


**Supplementary Figure 1. MALDI-TOF MS spectra of the mixed lipoprotein chemotypes in *B. subtilis* TXM1762 (Δ*hag P*$_{nat}$*-lit*, Ac-/lyso-LP).** Structures of parent tryptic peptides (*boxed* mass labels) corresponding to the *N*-terminus of the lipoprotein FrxB were assigned as either lyso-LP (*bold* mass labels) or Ac-LP (plus 42 Da, *mustard* arrows) according to **Fig. 3D**. Chemotype heterogeneity due to differences in total acyl chain lengths (minus 14 Da, *green* arrows) and sodiated adducts (*red* arrows) are indicated.

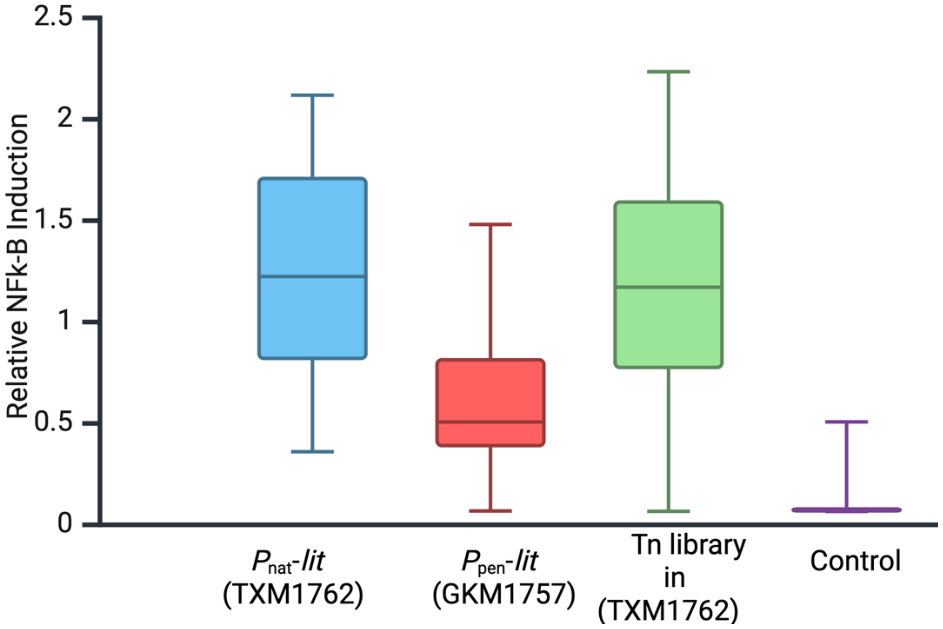


**Supplementary Figure 2. Transposon library screening for TLR2 activity.** Individual colonies from Tn libraries built in *B. subtilis* strain TXM1762 (*green*) were grown in microplates, heat killed, and used to challenge HEK-Blue hTLR2 NFκ-B inducible SEAP reporter cells. Approximately 6000 Tn mutants were screened in total, with hits defined as those with signal dropping at least 10-fold from the median observed with $P_{pen}$-*lit* (*red*, *n*=40 replicates). Tn insertion mutants that repeated in replicates using colony purified and CFU normalized bacterial cultures were mapped by inverse PCR as described in Materials and Methods. The parent strain TXM1762 (*blue*, *n*=40 replicates) and water control (*purple*, *n*=40) are shown for comparison.

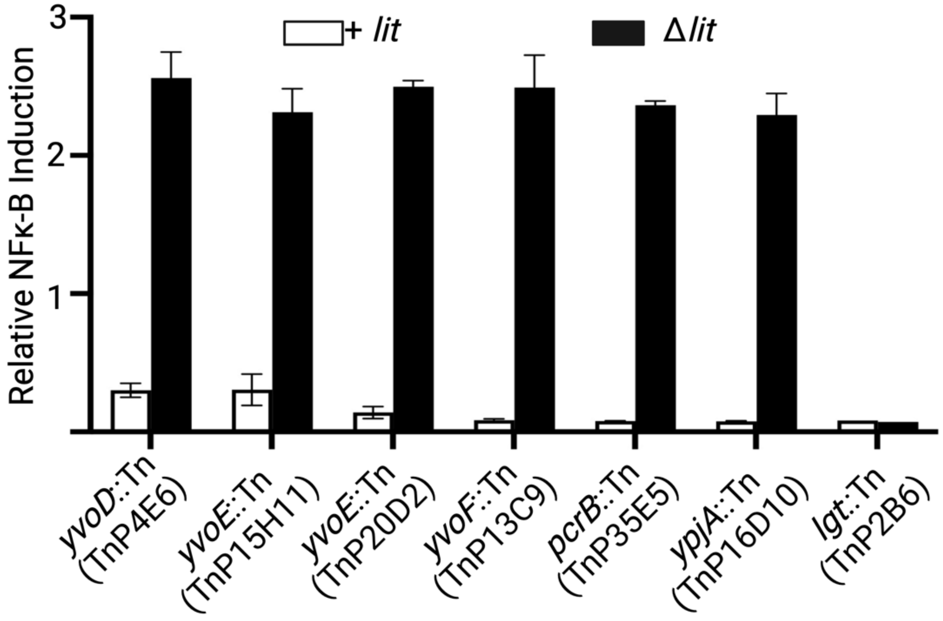


**Supplementary Figure 3. TLR2 stimulating activity of Tn insertion mutants.** Heat-killed aliquots of *B. subtilis* cultures grown to stationary phase were diluted 100-fold and applied to HEK-Blue hTLR2 NF-κB secreted alkaline phosphatase (SEAP) reporter cells. TLR2 activation was measured for both the indicated primary Tn mutant isolates expressing *lit* (TXM1762 background, Δ*hag* $P_{nat}$-*lit*, Ac-/lyso-LP, *shaded* bars) and from paired isogenic strains with the $P_{nat}$-*lit* cassette removed (*open* bars) to evaluate total lipoprotein levels. Data represents the average of three biological replicates.

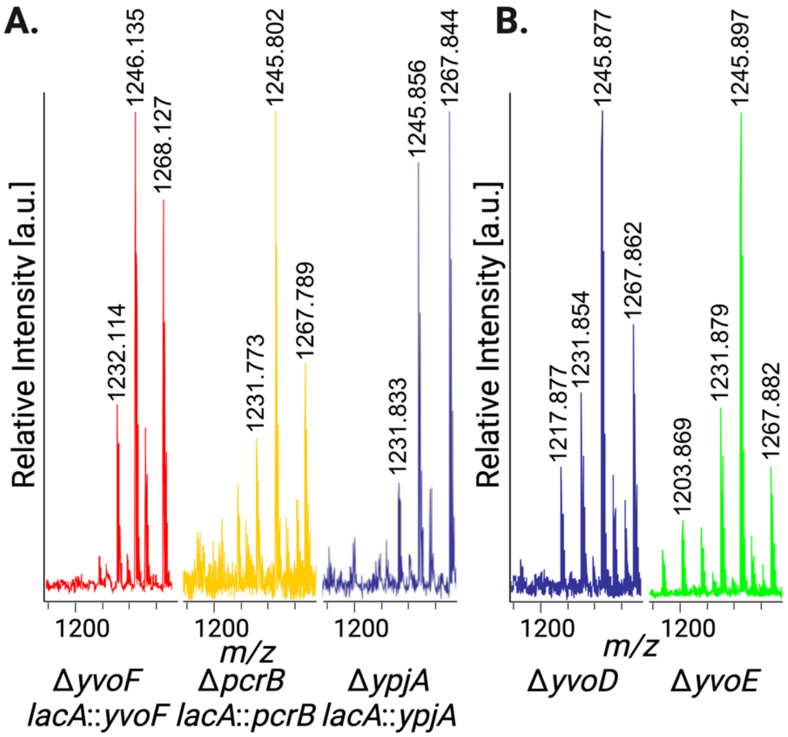


**Supplementary Figure 4. MALDI-TOF MS analysis of *B. subtilis* FrxB *N*-terminal lipopeptides. A.** Strains with essential Ac-LP pathway genes deleted were restored by ectopic back complementation [TXM1925 (Δ*yvoF*+*lacA*::*yvoF*), GKM1843 (Δ*pcrB*+*lacA*::*pcrB*), RW2143 (Δ*ypjA*+*lacA*::*ypjA*)]. **B.** Spectra were acquired for *B. subtilis* Δ*yvoD* (GKM1832) and Δ*yvoE* (GKM1833) to confirm Ac-LP biosynthesis and non essential roles in lipoprotein *N*-acetylation. The *N*-terminal tryptic lipopeptides from FrxB were assigned as indicated in **Fig. 3D**.

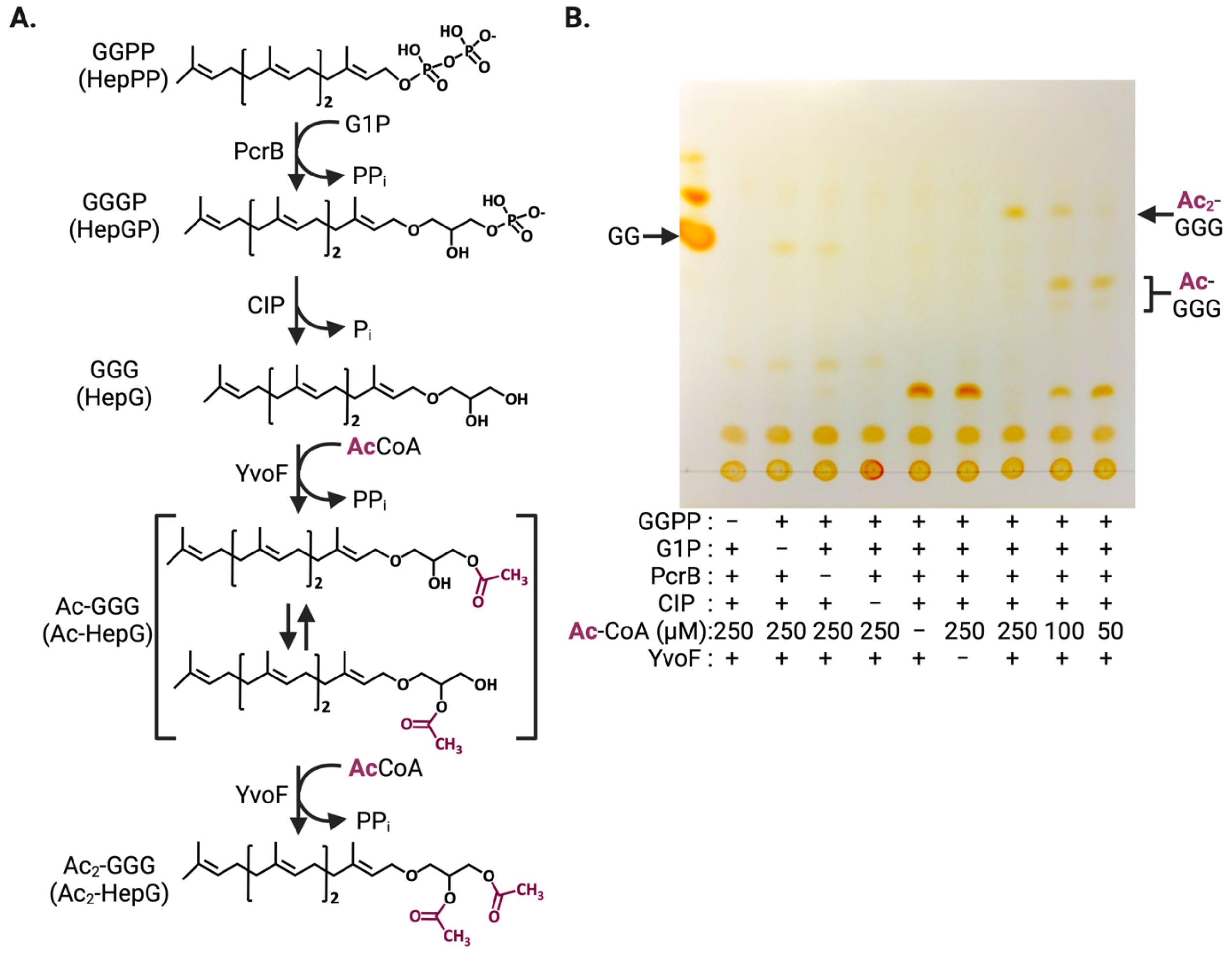


**Supplementary Figure 5. Synthesis of acetylated geranylgeranyl glycerol (Ac-GGG) carrier. A.** Commercially available geranylgeranyl pyrophosphate (GGPP, Cayman Chemical) with four isoprenoid units was condensed with glycerol 1-phosphate (G1P) using recombinant heptaprenylglyceryl phosphate synthase (PcrB), dephosphorylated using calf alkaline phosphatase (CIP), and loaded using acetyl-CoA (Ac-CoA) donor with heptaprenylglyceryl acetyl transferase (YvoF) as has been described (10). The position of the acetyl group on Ac-GGG is arbitrarily shown. The corresponding seven isoprenoid unit long substrates native in *B. subtilis* are indicated in parentheses. **B.** Reaction products were analyzed by TLC after incubation with (+) or without (-) the indicated components and visualized by iodine vapor staining. Both mono- (Ac-GGG at either C1 or C2 of glyceryl unit) and di- ($Ac_2$-GGG) acetylated carrier products were observed in a ratio dependent on the concentration of Ac-CoA. GG- geranylgeranyl alcohol diterpenoid standard.

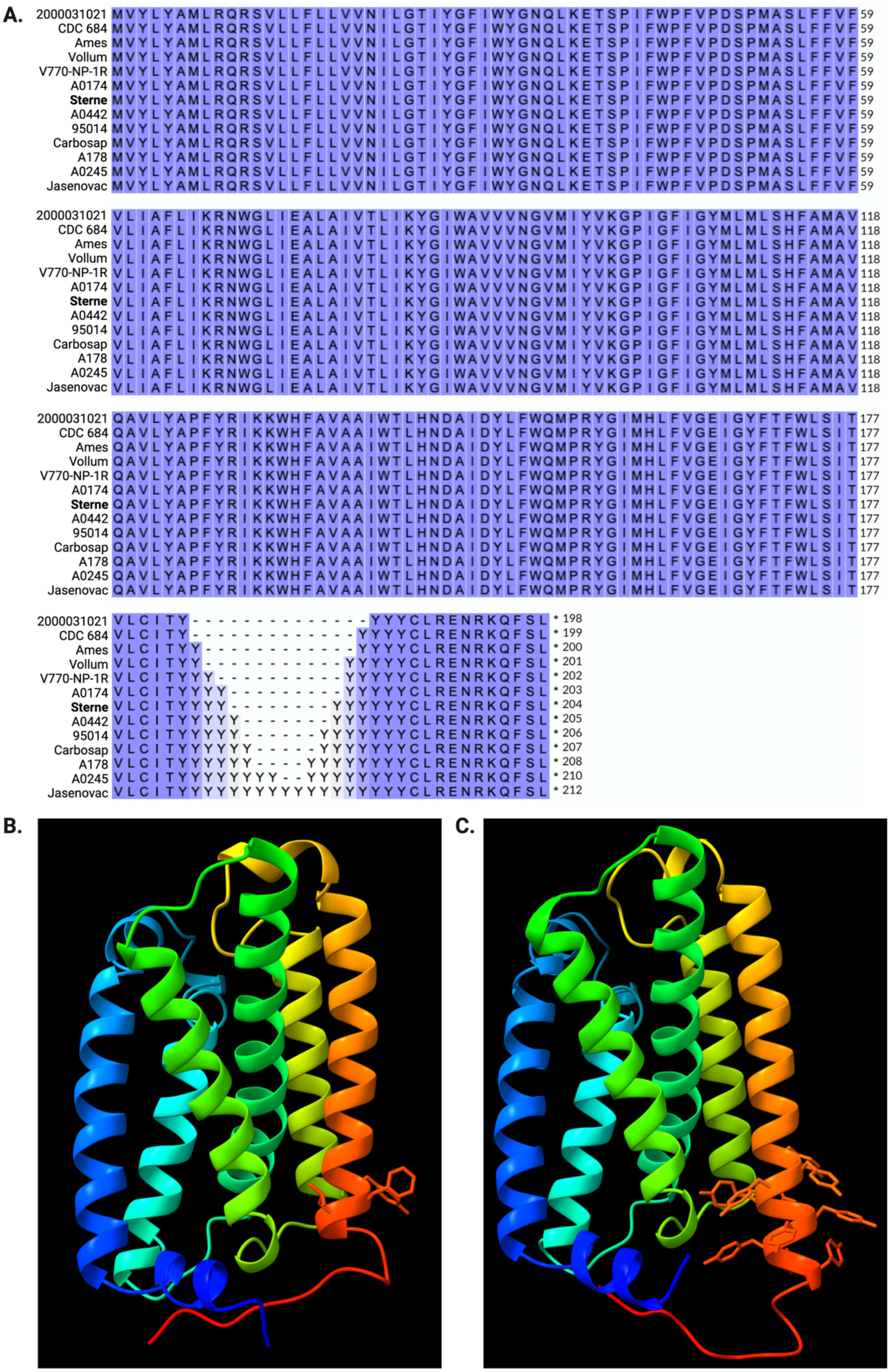


*B. subtilis*
LhaT (YpjA)

*B. anthracis*
LhaT (YpjA')

**Supplementary Figure 6. Sequence analysis and structure prediction of *B. anthracis* LhaT'(YpjA). A.** The amino acid sequences of LhaT'(YpjA) encoded in genomes of representative *B. anthracis* isolates were aligned with UniProt (11). Poly-Tyr insertions span from 4 to 18 Tyr residues, with Sterne having 10 Tyr residues. *B. anthracis* strain 2000031021(NCBI accession CP007618.1); CDC 684 (CP001215.1); Type strain Vollum (CP007666.1)**;** Ames (AE016879.1); V770-NP-1R (CP009598.1); A0174 (ABLT01000003.1); Sterne (CP009541.1); A0442 (ABKG01000003.1); 95014 (JANG01000015.1); Carbosap (JPIG01000023.1); A178 (CP076173.1); A0245 (DAOIRB010000003.1); Jasenovac (CP160831.1). **B.** The predicted structure of the *B. subtilis* LhaT(YpjA) lipoprotein *N*-acetyl transferase which has enzymatic activity *in vitro* (see **Fig. 3**). Side chains of the YFL (183-185) amino acids at the end of the sixth transmembrane $\alpha$-helical pass (*orange-red*) are shown. **C.** Predicted structure the *B. anthracis* Sterne LhaT'(YpjA) with a 10 Tyr residue long insertion (side chains shown) at the end of the sixth transmembrane $\alpha$-helical pass (*orange-red*). Substitution of the 10 Tyr residues with YFL restores the *in vitro* activity of *B. anthracis* (see **Fig. 5**). Structures were predicted using AlphaFold 3 (12).

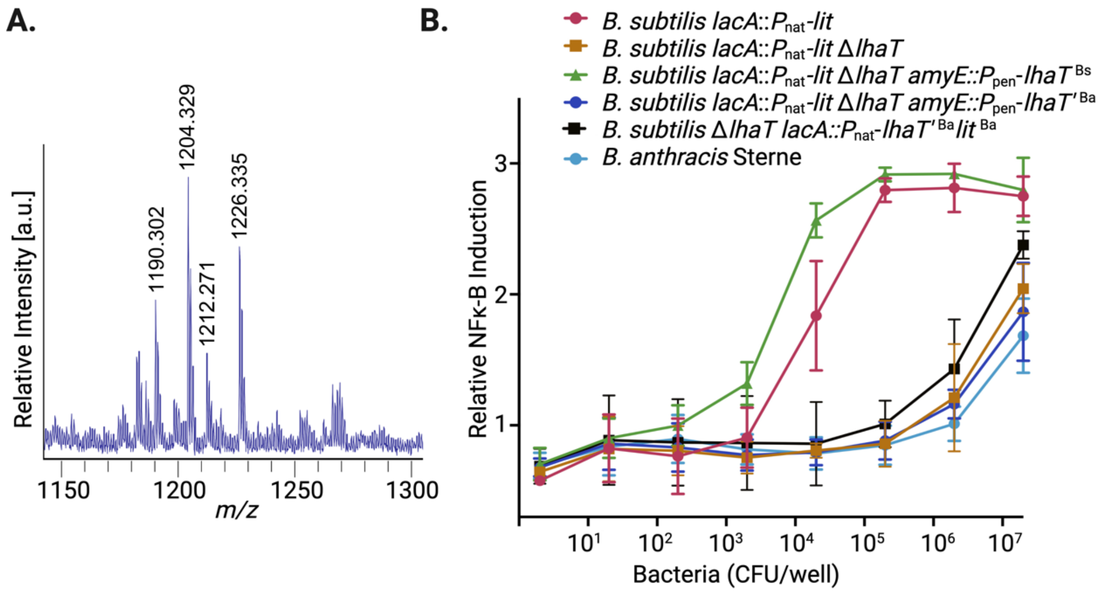


**Supplementary Figure 7. LhaT'(YpjA) from *B. anthracis* is non-functional. A.** MALDI-TOF MS spectra of FrxB *N*-terminal tryptic lipopeptides (mass peaks assigned as indicated in **Fig. 3D**) isolated from *B. subtilis* RW2625 (Δ*ypjA amyE::$P_{pen}$-$ypjA^{Ba}$*, DA-LP). The *B. anthracis* LhaT' orthologue is unable to form Ac-LP and replace the function of LhaT from *B. subtilis*. **B.** HEK-Blue hTLR2 reporter cells were challenged with heat-killed extracts of *B. subtilis* expressing *lit* (TXM1762 *lacA*::$P_{nat}$-*lit*), with *lhaT* also deleted (TXM1939 *lacA*::$P_{nat}$-*lit* Δ*lhaT*), and after back complementation with *lhaT* from *B. subtilis* (RW2205 *lacA*::$P_{nat}$-*lit* Δ*lhaT amyE::$lha^{Bs}$*) or *B. anthracis* (RW2535 *lacA*::$P_{nat}$-*lit* Δ*lhaT amyE::$lhaT^{Ba}$*). The cassette from the *B. anthracis* *$lhaT'^{Ba}$*-*$lit^{Ba}$* operon under its native promotor (RW2673 Δ*lhaT lacA::$P_{nat}$-$lhaT'^{Ba}$ $lit^{Ba}$*) shows low TLR2 activity comparable to *ΔlhaT* (TXM1939) indicating *$lhaT'^{Ba}$* cannot compete with *lit* for DA-LP substrate. Heat-killed extracts of wildtype *B. anthracis* Sterne also show low TLR2 activation, consistent with a lyso-LP population. Data represents the average of three biological replicates.

## REFERENCES


1. Burby PE, Simmons LA. 2017. MutS2 Promotes Homologous Recombination in *Bacillus subtilis*. J Bacteriol 199.
2. Santiago M, Matano LM, Moussa SH, Gilmore MS, Walker S, Meredith TC. 2015. A new platform for ultra-high density *Staphylococcus aureus* transposon libraries. BMC Genomics 16:252.
3. Hartl B, Wehrl W, Wiegert T, Homuth G, Schumann W. 2001. Development of a new integration site within the *Bacillus subtilis* chromosome and construction of compatible expression cassettes. J Bacteriol 183:2696-9.
4. Yuan G, Wong SL. 1995. Regulation of groE expression in *Bacillus subtilis*: the involvement of the sigma A-like promoter and the roles of the inverted repeat sequence (CIRCE). J Bacteriol 177:5427-33.
5. Meredith TC, Swoboda JG, Walker S. 2008. Late-stage polyribitol phosphate wall teichoic acid biosynthesis in *Staphylococcus aureus*. J Bacteriol 190:3046-56.
6. Wang H, Claveau D, Vaillancourt JP, Roemer T, Meredith TC. 2011. High-frequency transposition for determining antibacterial mode of action. Nat Chem Biol 7:720-9.
7. Charpentier E, Anton AI, Barry P, Alfonso B, Fang Y, Novick RP. 2004. Novel cassette-based shuttle vector system for gram-positive bacteria. Appl Environ Microbiol 70:6076-85.
8. Dempwolff F, Sanchez S, Kearns DB. 2020. TnFLX: a Third-Generation mariner-Based Transposon System for *Bacillus subtilis*. Appl Environ Microbiol 86.
9. Armbruster KM, Komazin G, Meredith TC. 2020. Bacterial lyso-form lipoproteins are synthesized via an intramolecular acyl chain migration. J Biol Chem 295:10195-10211.
10. Linde M, Peterhoff D, Sterner R, Babinger P. 2016. Identification and Characterization of Heptaprenylglyceryl Phosphate Processing Enzymes in *Bacillus subtilis*. J Biol Chem 291:14861-70.
11. UniProt C. 2025. UniProt: the Universal Protein Knowledgebase in 2025. Nucleic Acids Res 53:D609-D617.
12. Abramson J, Adler J, Dunger J, Evans R, Green T, Pritzel A, Ronneberger O, Willmore L, Ballard AJ, Bambrick J, Bodenstein SW, Evans DA, Hung CC, O'Neill M, Reiman D, Tunyasuvunakool K, Wu Z, Zemgulyte A, Arvaniti E, Beattie C, Bertolli O, Bridgland A, Cherepanov A, Congreve M, Cowen-Rivers AI, Cowie A, Figurnov M, Fuchs FB, Gladman H, Jain R, Khan YA, Low CMR, Perlin K, Potapenko A, Savy P, Singh S, Stecula A, Thillaisundaram A, Tong C, Yakneen S, Zhong ED, Zielinski M, Zidek A, Bapst V, Kohli P, Jaderberg M, Hassabis D, Jumper JM. 2024. Accurate structure prediction of biomolecular interactions with AlphaFold 3. Nature 630:493-500.
